# Hardware-in-loop implementation of an adaptive MPPT controlled PV-assisted EV charging system with vehicle-to-grid integration

**DOI:** 10.1038/s41598-025-12508-3

**Published:** 2025-08-05

**Authors:** Surabhi Singh, Hari Om Bansal

**Affiliations:** Present Address: Department of Electrical and Electronics Engineering, Birla Institute of Technology and Science, Pilani, Pilani Campus, Vidya Vihar, Pilani, Rajasthan 333031 India

**Keywords:** Electric vehicles, Hybrid MPPT, Vehicle-to-grid, PV-grid integration, Hardware–Hardware-in-loop, Energy science and technology, Engineering

## Abstract

The penetration of electric vehicles (EVs) into society needs extensive charging infrastructure. The existing charging system solely depends on the grid supply, which is essentially fossil fuel-dependent and leads to carbon emissions and environmental pollution. This can be minimized by incorporating renewable energy into the charging grid. This article presents a charging scheme combining photovoltaic (PV) and grid, offering a clean and dependable charging plan to sustain green transport. The proposed work presents the modelling and controlling a 10 kW EV charging/discharging framework integrating PV and grid. This work has multi-fold objectives: i) the development of an intelligent hybrid maximum power point tracking (MPPT) strategy, ii) the design of a fuzzy logic controlled bidirectional charger, iii) the setup of a PV-grid integrated charging system, and iv) the implementation of vehicle-to-grid (V2G) operation. The proposed charging system utilizes PV power and seamlessly switches to grid power whenever required. Since the performance of the PV source is affected by varying temperatures and irradiance, MPPT methods are needed to extract maximum power from the PV source. This paper developed and compared perturb and observe (P&O), Particle swarm optimization (PSO), and hybrid PSO + Adaptive neuro-fuzzy inference system (ANFIS) based algorithm for MPPT. The findings indicate that the PSO + ANFIS-driven method offers the highest tracking efficiency of 99.5%. This algorithm is also tested under dynamic partial shading conditions (PSC) to ensure robustness, and it led to achieving fast convergence and high efficiency despite multiple power peaks. In addition, the designed bidirectional charging system maximizes solar energy collection, minimizes the charging cost, and improves grid stability through demand balancing. The overall system is validated in a hardware-in-loop real-time environment through FPGA-based OPAL-RT.

## Introduction

The increasing environmental pollution due to conventional internal combustion engine (ICE) vehicles has resulted in global warming^1^. In the last decade, concern about the ecological impact of carbon-emitting vehicles has increased tremendously^2^. To address this problem, there is a shift towards adoption of EVs which minimizes the tail pipe emission. Globally, countries focusing on manufacturing and developing EVs to reduce air pollution^3,4^. The sales of EVs highly depends on storage battery technology and availability of the charging infrastructure. According to the International Energy Agency (IEA), EV sales reached 14 million in 2023 across the globe, posting a 35% increment compared to year 2022 numbers, showing an accelerated shift towards e-mobility^5^. Further, to tackle this nonlinear EV load increasing the, the grid system needs significant improvement and intelligence^6^. Sudden penetration of EVs and their uncoordinated charging may lead to stress on the power grid and blackouts, therefore, optimal charging of EVs at a time is required to deal with this situation. Shortly, rapid charging will become a norm to compete with the conventional vehicles. Also, bidirectional charging should be integrated so EVs can act as distributed generators while connected to the power grid. Many successful pilot projects in countries like the Netherlands, Japan, and United Kingdom have already proved the grid-support potential of V2G-enabled EVs in smoothing peak loads and improving demand-side flexibility^7^. This will result in the enhancement of grid stability and reliability. In addition, renewable energy integrated advanced charging infrastructure will further enhance the efficiency and sustainability of the power network. Furthermore, an optimally integrated V2G system improves peak power demand management and reliability for the upcoming smart grids. Smart grid architectures that include V2G-enabled EVs are also being considered in IEEE 2030 standards for modernization of the grid^8^.

Effective power flow control in renewable energy integrated systems demands sophisticated converters and control strategies^9^. Traditional methods, including typical PI controllers^10^, are unable to provide optimal performance in terms of stability and dynamic response under varying operating conditions. Particularly in dual active bridge (DAB) converters, conventional methods are more prone to overshoots and non-adaptability, which result in inferior charging efficiency and performance of EVs^11^. The DAB converter, which is an isolated bidirectional DC-DC converter based on high-frequency transformers, is commonly employed in EV fast-charging and V2G applications because it offers bi-directionality in the path of power flow and also provides galvanic isolation between the input and output sides. The limitations of linear control techniques in nonlinear and bidirectional power conversion scenarios i.e. charging/discharging, have been emphasized in a number of studies^12^. Therefore, there is a growing trend toward the application of intelligent control techniques that are capable of managing system uncertainties and parameter fluctuations^13^. Various researchers are progressively employing intelligent control techniques to improve the real-time efficiency and flexibility of power electronic systems in smart charging infrastructure^14–16^. However, most of these studies remain confined to simulation environments, and real-time hardware validation is often missing.

The operation of PV systems depends on environmental conditions like irradiance and temperature, so utilizing the maximum power available under diverse conditions is vital^17^. Researchers have studied conventional MPPT methods that are simple and easy to implement, such as incremental conductance (INC) and P&O^18^. However, these methods have limitations such as slow tracking speed, steady-state oscillations, and poor performance in rapidly varying conditions, therefore intelligent methods like fuzzy logic control, artificial neural networks (ANN), genetic algorithms (GA), and machine learning-based approaches were proposed to overcome these limitations^19–22^. Fuzzy logic improves system uncertainty handling capabilities, whereas ANNs can learn nonlinear data relationships. PSO, a population-based metaheuristic algorithm, has been found to possess high potential in MPP optimization owing to its feature of local minima avoidance and high convergence speed^23–25^.

While conventional methods such as P&O and INC are simple, their performance deteriorates under dynamic or rapidly changing irradiance conditions. To address this, several improved versions have been developed. A ripple-free boundary control-based MPPT strategy was proposed in^26^, offering precise tracking with minimal voltage oscillation and validated through both simulation and experimental platforms. In^27^, an adaptable step-size P&O algorithm was introduced for standalone PV systems integrated with battery storage, enhancing convergence speed and efficiency. Similarly^28^, proposed a theta-based MPPT method with an adjustable step-size mechanism that demonstrated high accuracy under varying atmospheric conditions. A low-cost, indirect MPPT strategy using a proportional–integral–derivative (PID) controller was developed in^29^, showing improved efficiency with reduced sensor requirements. These enhanced classical methods serve as a useful bridge between basic MPPT logic and more advanced, intelligent control strategies.

Partial shading conditions (PSC) present one of the most significant challenges in PV systems, leading to multiple local maxima in the power–voltage (P–V) curve. To overcome this, Siddique et al.^30^ introduced an adapted perturb and observe (APO) MPPT algorithm integrated with model predictive control (MPC), which achieved a tracking efficiency of 99.46% with fast convergence and minimal oscillation, validated using experimental hardware. Another study^31^ utilized a reference current–based MPC strategy for improved global maximum power point (GMPP) prediction under dynamically varying conditions. In^32^, a grey wolf optimization (GWO) technique was applied for MPPT under complex PSC profiles and shown to outperform conventional approaches. Siddique et al.^33^ also implemented an INC algorithm with an integral regulator in a grid-connected PV setup to enhance steady-state accuracy. A comprehensive review^34^ summarized trends in MPPT algorithm development, emphasizing hybrid methods, intelligent control, and the growing importance of real-time validation. These studies collectively highlight the need for adaptive, high-efficiency MPPT controllers that perform reliably under both uniform and shaded conditions.

Enhancing charging system efficiency using intelligent MPPT techniques is now becoming an area of interest, many such studies are narrated in Table 1. Ahmad et al. proposed a dual ANN controller for a single PV system to estimate irradiance and temperature^35^. The proposed system is integrated with a micro-grid, and the results using MATLAB Simulink showed improved efficiency and fast response time. However, it does not include real-time validation, grid integration, or EV charging capability. Yakkob et al. presented an INC based MPPT that targeted lowering the hardware cost by reducing the number of current sensors^36^. The study aimed to charge Li-ion batteries and proposed MPPT to achieve higher efficiency and fast response, but it is limited to standalone PV and battery. It has no grid, bidirectional power transfer, or EV application.

**Table 1:** Comparison of proposed work with some previous researches.

Reference and year of publication	MPPT technique used	Bi-directional converter used	Control strategy	V2G implementation	Real-time validation
^35^**, 2025**	Dual ANN for PV	Class c bidirectional dc-dc converter for battery and super-capacitor	MPPT control, maintains DC Bus voltage regulation at 380 V, hybrid control	No. It utilizes DC nano grid with single, PV battery and super-capacitor, with unidirectional power flow	No
^36^**, 2025**	Single INC method for PV	Unidirectional boost converter	Battery charging control between PV and battery	No	Yes, but Processor in loop (PIL) testing using
^37^**, 2024**	ANN-PSO MPPT	No bidirectional converter mentioned, three phase inverter for grid interface	Fuzzy control for charge/discharge	Yes, V2G, grid connected EVs with PV	No
^38^**, 2021**	Hybrid WO + PS tuned ANFIS-based INC MPPT	No	Active and reactive power control for grid interaction	No	No
^39^**, 2025**	RTHA to tune ANN for MPPT	Yes, multiport bidirectional converter	Hybrid energy management in PV, battery, super-capacitor	No	No
**Proposed work**	PSO + ANFIS MPPT	DAB converter	Bidirectional power flow from battery to grid, fuzzy control for DAB	Yes	Yes

Nouri et al. proposed a 105.7 kW PV system integrated with the grid, utilizing an intelligent ANN-PSO-based MPPT and an FLC for EV battery charging^37^. The battery charge converter is a bidirectional buck-boost converter connected to the DC bus, and it also comprises a three-phase inverter with voltage source control and an LCL filter for grid connectivity. Although, the proposed algorithm was fast and minimized oscillation under varying irradiance, and the FLC intelligently controlled V2G operations however, no real-time validation is performed. Hai Tao et al. implement a hybrid whale optimization (WO) with a pattern search (PS) algorithm. It utilizes an ANFIS-based INC with PI control for grid integration with PV. The work focuses only on PV and the grid. The entire work is simulated in MATLAB Simulink^38^. Richard et al. utilized an red-tailed hawk optimized (RTHA)-ANN MPPT for a PV-battery-super-capacitor and EV system with internal bidirectional flow but without grid support^39^. While these studies are informative regarding advanced MPPT and control algorithms, they are not fully PV–EV–grid coordinated or real-time adaptive under highly variable conditions. While these studies are informative regarding advanced MPPT and control algorithms, they are not fully PV–EV–grid coordinated or real-time adaptive under varying conditions.

Though these strategies demonstrate significant advancements in MPPT control, they are mainly developed for isolated PV or PV–battery systems and are not optimized for dynamic bidirectional power flow scenarios, V2G infrastructure, or real-time environmental dynamics. For comparison, this work presents a full-scale, real-time coordinated energy management approach for PV–EV–grid systems, verified with HIL simulation. The proposed system is realized on the OPAL-RT real-time simulation platform and utilises a new hybrid PSO + ANFIS-based MPPT controller, with a bidirectional DAB converter and smart switching logic to handle both G2V and V2G power flows. A hybrid PSO + ANFIS MPPT controller is utilized, which is able to maintain high tracking efficiency (99.5%) and low steady-state error (< 0.05%) in the presence of sharp irradiance variations (tested with 1000, 800, 600, 400 and 200 W/m^2^). Most importantly, the system dynamically allocates PV and grid power so that whenever solar power is inadequate, the grid supplies seamlessly to ensure uninterrupted EV charging and load support. This intelligent hybrid routing approach, coupled with real-time switching logic and bidirectional DAB control, provides for solid and adaptive power transmission under a variety of operating conditions which are not fully covered in prior research efforts.

The proposed study tries to minimize these issues and contributes to advancing renewable energy based EV charging systems by addressing key challenges in energy management, control strategies, and real-time implementation. A brief comparison of key features of previous researches with the proposed work is given in Table 1.

This paper proposes a fuzzy (type-2) tuned PI controller for the DAB converter integrated with a PV system using hybrid, PSO + ANFIS based MPPT. The proposed system enables efficient bidirectional charging, allowing energy flow from the PV and/or grid to charge the EV battery and also power flow from vehicle to grid under varying solar and load conditions. The major contributions of this study include:

1. Developing a PSO + ANFIS based MPPT algorithm for optimal power extraction from PV system and its comparison with P&O and PSO algorithm shows the betterment in power extraction.
2. Developing a hybrid, PV and grid system for energy management to reduce day time burden on grid. This system intelligently switches between PV and grid to charge the EV.
3. The proposed system supports bidirectional energy transmission between the utility grid and the EV battery (i.e. G2V and V2G). It reduces grid stress and improves energy resilience enabling EV as a distributed energy resource during peak demand.
4. Hardware-in loop implementation of the proposed integrated system in real-time using Opal-RT, demonstrating its capability to handle dynamic energy conditions and ensure stable operation.

**Fig. 1:**
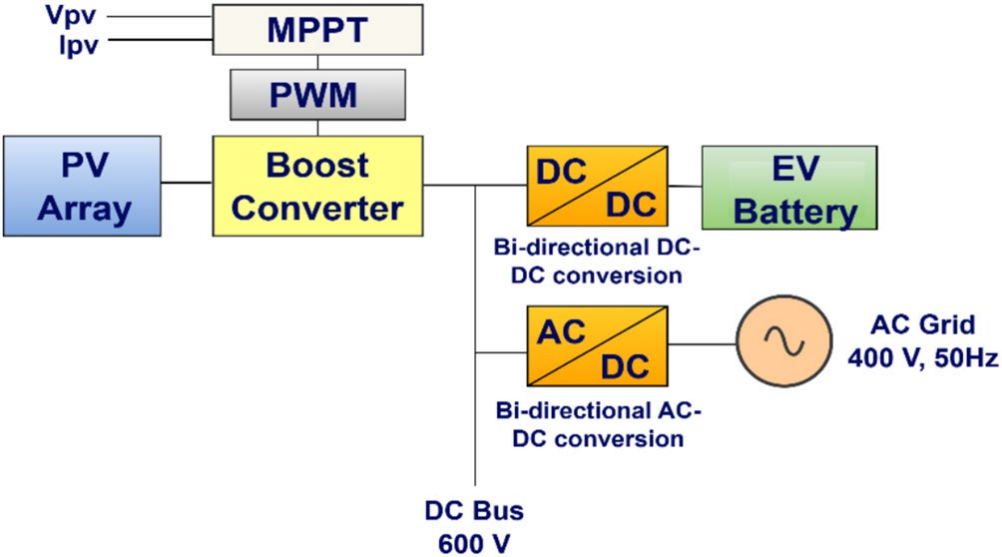
Proposed architecture of EV’s charging and discharging with PV and the grid.

Figure 1. depicts the proposed system setup; a PV system with an MPPT-enabled boost converter is interfaced at the DC link; a bidirectional DC-DC converter connecting the EV battery to the grid and PV source. The converter controls the charging and discharging operations of the battery. An AC-DC converter feeds power to DC-DC converter, functioning as a rectifier during charging and as an inverter during discharging of EV battery, enabling power injection from vehicle to grid under source converter control. Depending on the mode of operation, the setup allows PV source to supply power either to the grid or directly to the EV battery.

This paper is structured as follows: Section"Proposed system description"describes the proposed system, Section"Methods employed in the proposed study"outlines the methodology used and MPPT techniques employed in the proposed work, Section"Results:"consists of results and discussions carried out in the paper, and finally, Section"Conclusion"consists of the conclusion part of the paper.

## Proposed system description

The architecture of proposed system is shown in Fig. 1. A brief description of its major components is given below.

### PV with integrated boost converter

**Fig. 2:**
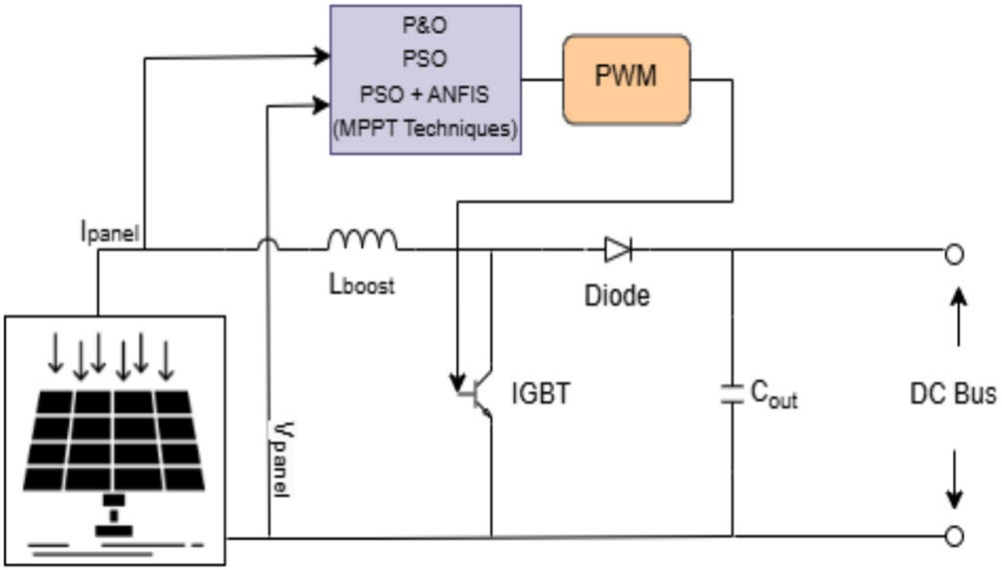
PV module network linked with boost converter.

PV module is interfaced with DC bus using a boost converter to maintain its voltage level that matches with the DC bus voltage (600 V). This setup stabilizes and controls power at the DC bus. The boost converter is regulated through an MPPT algorithm for maximum energy harvesting from the PV source, regardless of ambient conditions. By regulating the output voltage, the boost converter enables the smooth transfer of solar power to the EV battery via the bidirectional DC-DC converter or to the grid via an AC-DC converter based on the mode of operation. A schematic diagram of PV module linked with boost converted is depicted in Fig. 2.

**Fig. 3:**
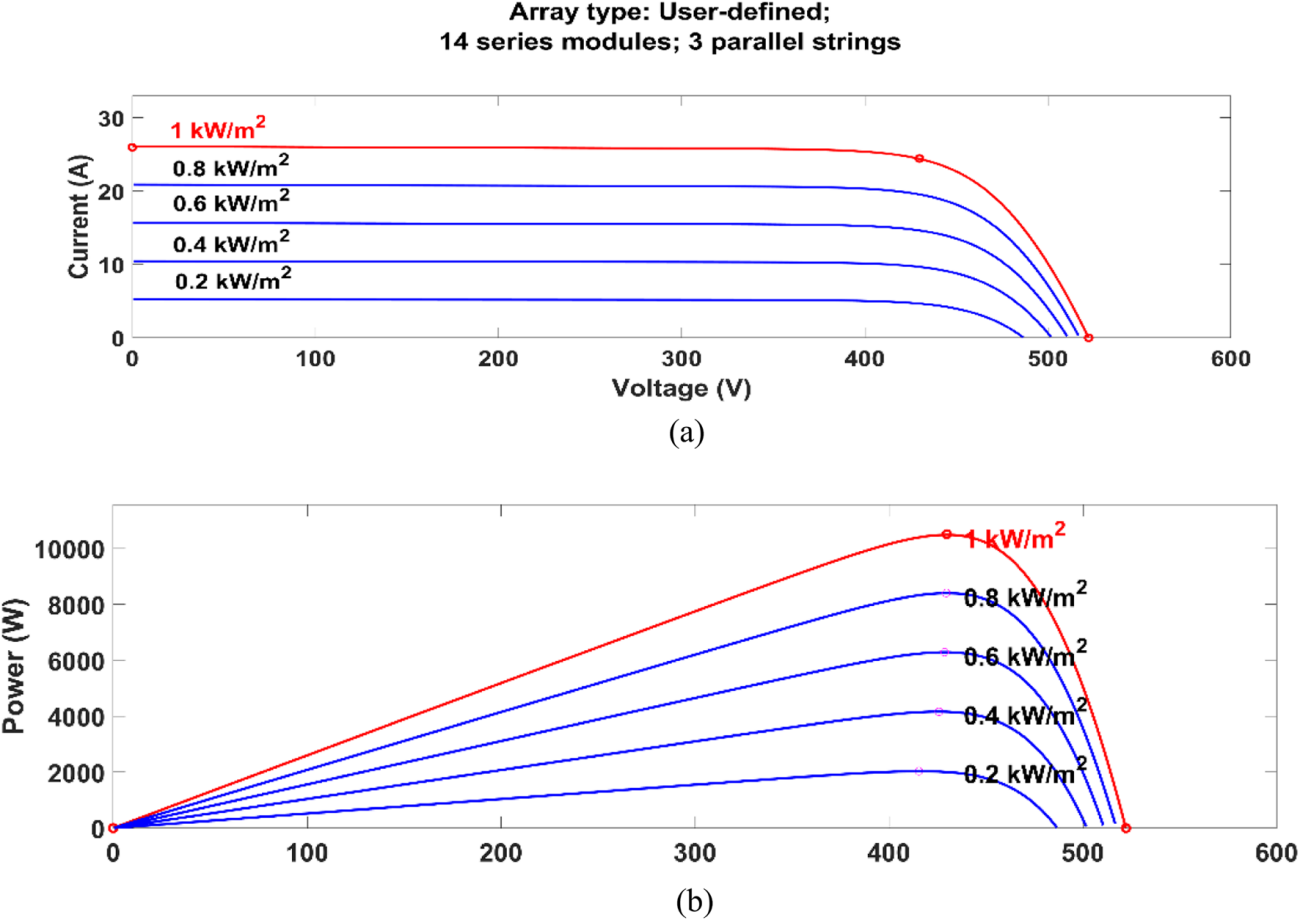
The current vs voltage (**a**) and power vs voltage (**b**) graphs with respect to irradiance change.

The PV array is designed using the ratings given in Table 2. The maximum energy extraction point is shown in Fig. 3, where the current vs voltage and power vs voltage graphs are demonstrated for selected PV modules for the proposed work.

**Table 2:** PV panel used in the system (Electrical properties).

Parameters	Values
Cell per module	60
Maximum power (P_max_​)	250.205W
Voltage at P_max_​	30.7
Current at P_max_​	8.15
V_oc_	37.3
I_sc_	8.66
Temperature coefficient (V_oc_)	−0.36901
Temperature coefficient (V_oc)_	0.086998

### Three phase bi-directional converter

**Fig. 4:**
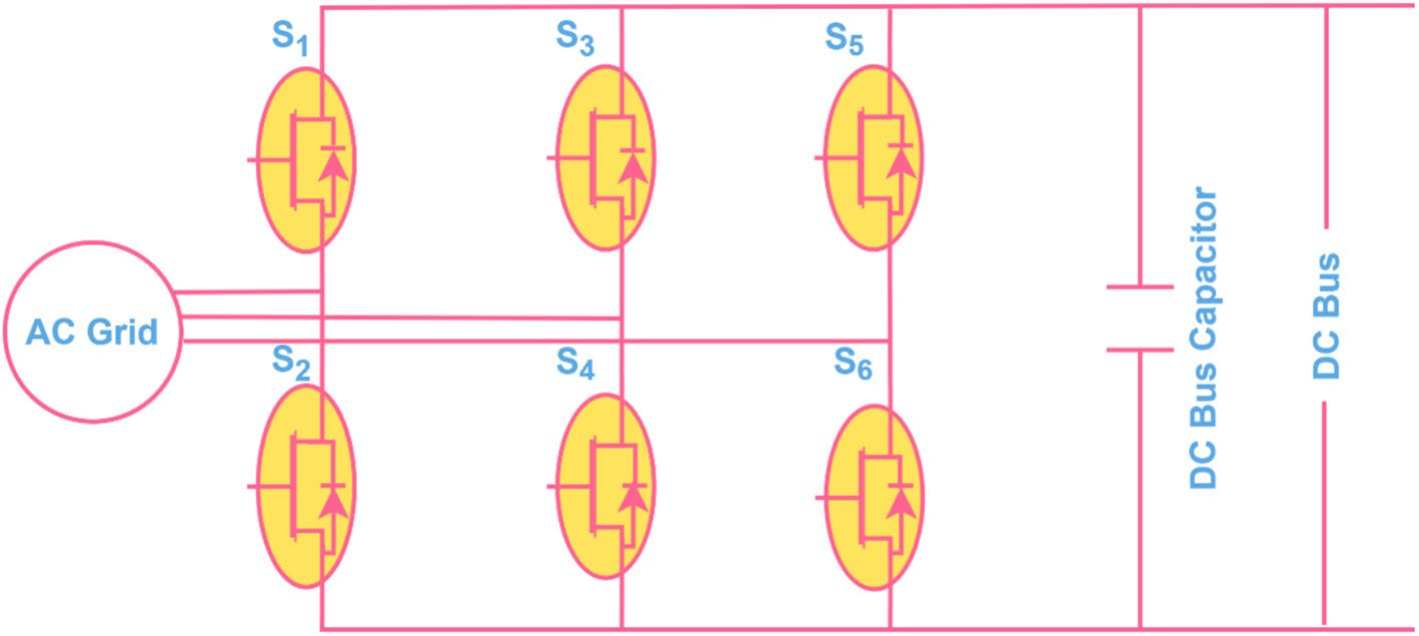
3-phase bidirectional source converter for DC-AC and AC – DC power conversion.

The source converter shown in Fig. 4, generally acts as a bidirectional voltage source converter, which connects the grid with the DC Bus. It maintains DC bus voltage and synchronizes with the grid using a phase-locked loop. Further, it enables G2V and V2G modes; in G2V mode, it works as a rectifier, and in V2G mode, it acts as an inverter.

### Dual active bridge converter

**Fig. 5:**
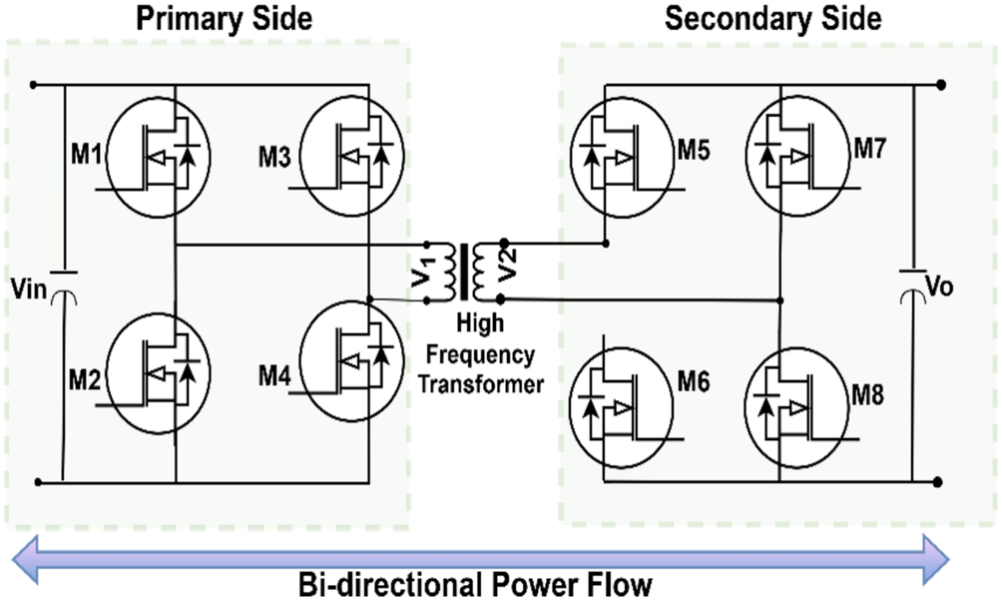
Schematic of DAB converter.

The conventional DC-DC bidirectional buck-boost or fly-back converters are non-isolated, limiting their capacity for galvanic isolation between the input and output power transfer^40^. The absence of isolation may lead to safety, voltage regulation, and system integration difficulties, particularly when interfacing with renewable energy sources and energy storage systems. Also, these converters have greater conduction losses and comparatively lesser efficiency at high power ratings. However, the DAB converter provides galvanic isolation, which enhances safety and facilitates better voltage regulation efficiency. The ability of the DAB to switch at high frequencies minimizes the size of passive components and improves its performance in dynamic power flow situations. This makes the DAB converter a better option for the proposed system, where efficiency, flexibility, and high-frequency operation are essential. The DAB converter circuit using the primary and secondary bridges is illustrated in Fig. 5. When each bridge duty cycle is adjusted to 50%, square waves are generated at both transformer ends, respectively. To regulate the power flow direction between the two connected DC sources, the two square waves undergo a phase shift of angle Ø.

#### Control structure for DAB

**Fig. 6:**
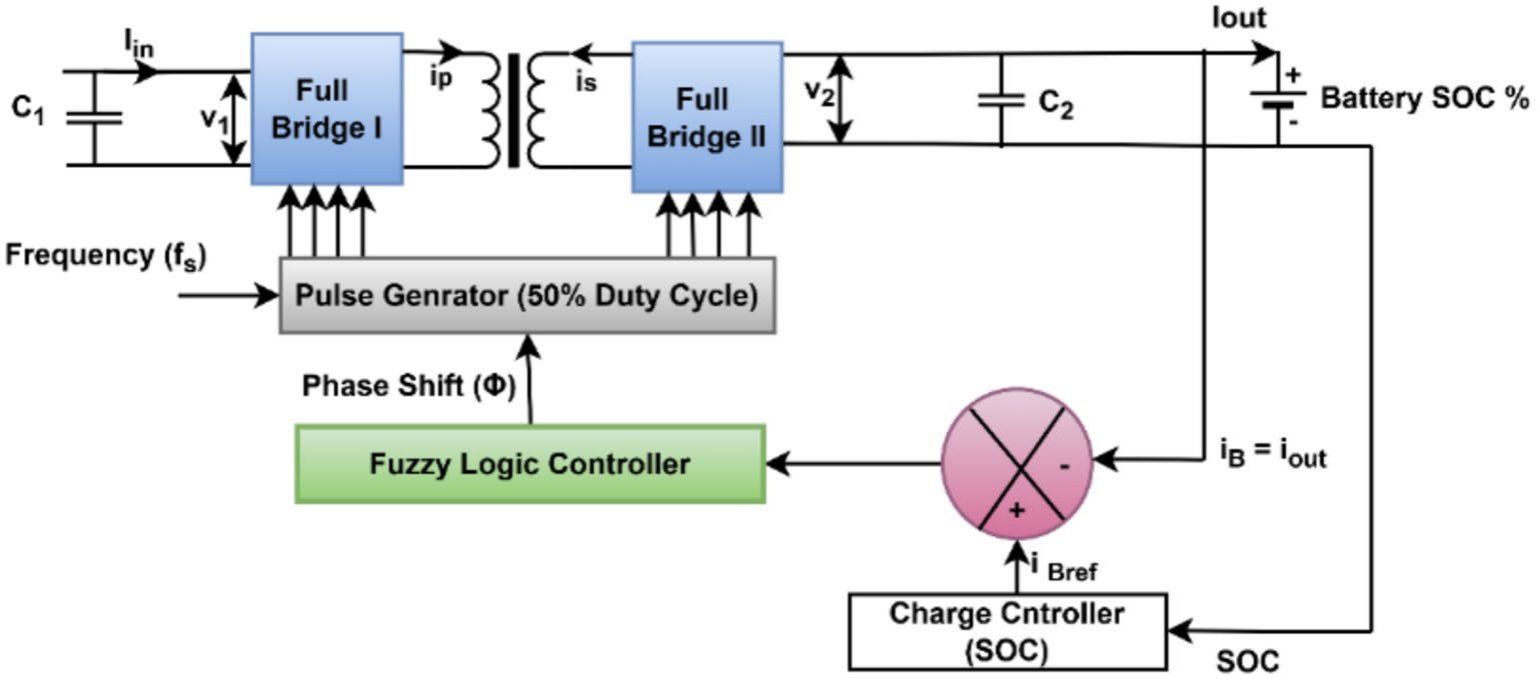
DAB controller proposed in the integrated structure.

The DAB converter manages the bidirectional battery charging and discharging; in Fig. 6, it is shown that the battery is connected to the DAB converter. In the proposed work, an intelligent control approach is developed using a fuzzy controller to regulate the power transfer of the DAB converter for bidirectional battery charging and discharging by dynamically adjusting the phase shift angle. The motivation behind employing a fuzzy controller stems from its inherent ability to handle non-linearities, parameter uncertainties, and dynamic variations in real time, which are common in renewable-integrated charging infrastructures.

To improve robustness against noise modelling uncertainty, fuzzy type-2 Sugeno fuzzy logic controller. The type-2 system also introduces a Footprint of Uncertainty (FOU) in the membership functions, allowing it to capture uncertainties better and deliver improved performance under fluctuating load and source conditions.

### Active and reactive power PQ control (For G2V and V2G)

**Fig. 7:**
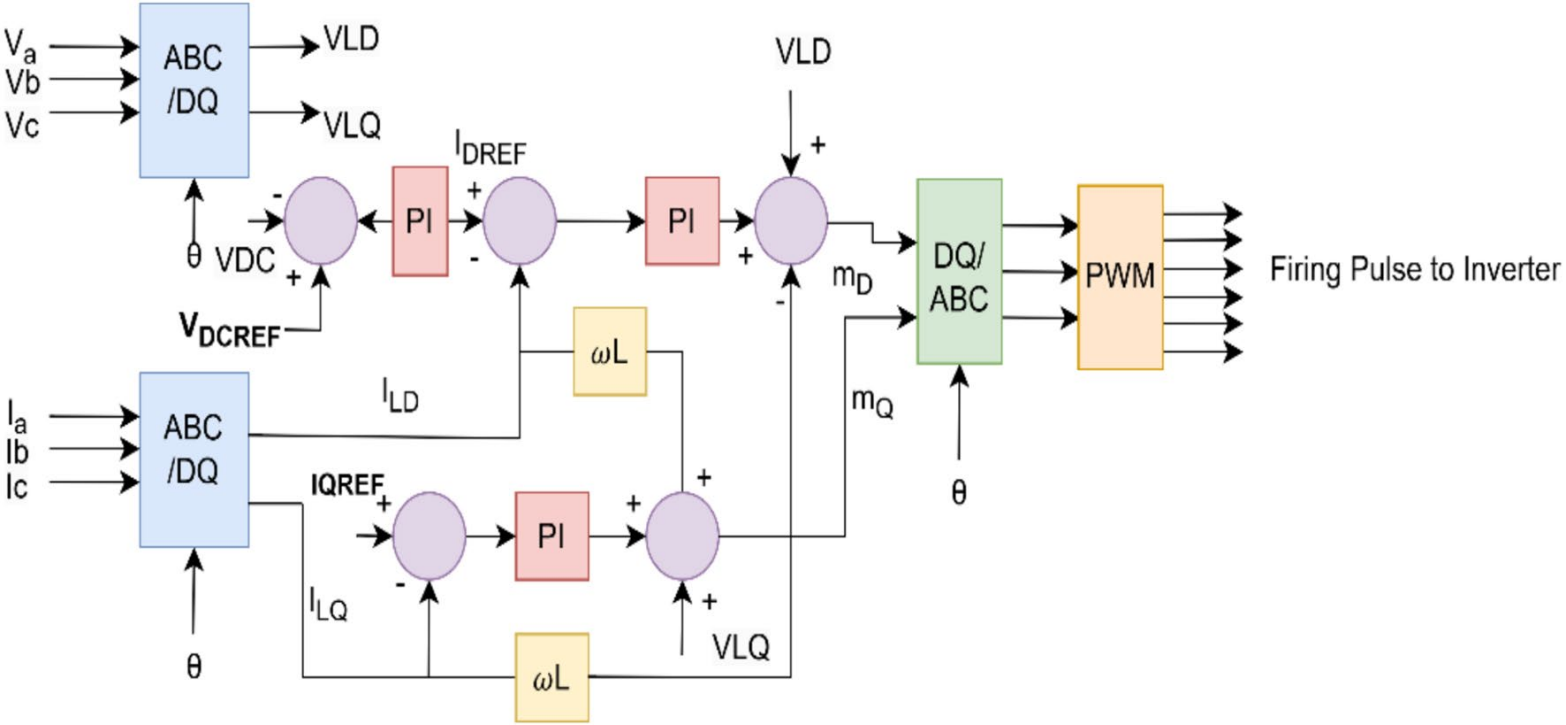
PQ control structure.

The proposed scheme uses the PQ control shown in Fig. 7, for power flow management between the grid and the DC bus. Its function is to maintain DC voltage stability and regulation of grid voltage. It independently regulates active and reactive power and enables bidirectional flow between the grid and the DC bus. It is implemented in a synchronous frame by decoupling power components by transforming the 3-phase signals into direct and quadrature axes using the Clarke and Parks transformation technique.

## Methods employed in the proposed study

In the beginning, this section outlines the various MPPT techniques, namely P and O, PSO and PSO + ANFIS based algorithm. The specific use of the MPPT algorithm is to check and modify the duty cycle of the boost converter and ensure the generation of maximum power output from the PV Panel. Later, the controller design for DAB converter used for bidirectional EV charging/discharging is presented.

### MPPT technique

#### Description of The MPPT system

The MPPT scheme integrated into the proposed architecture is used to ensure the maximum power extraction from the PV module regardless of changing environmental conditions.

#### PV cell modelling

**Fig. 8:**
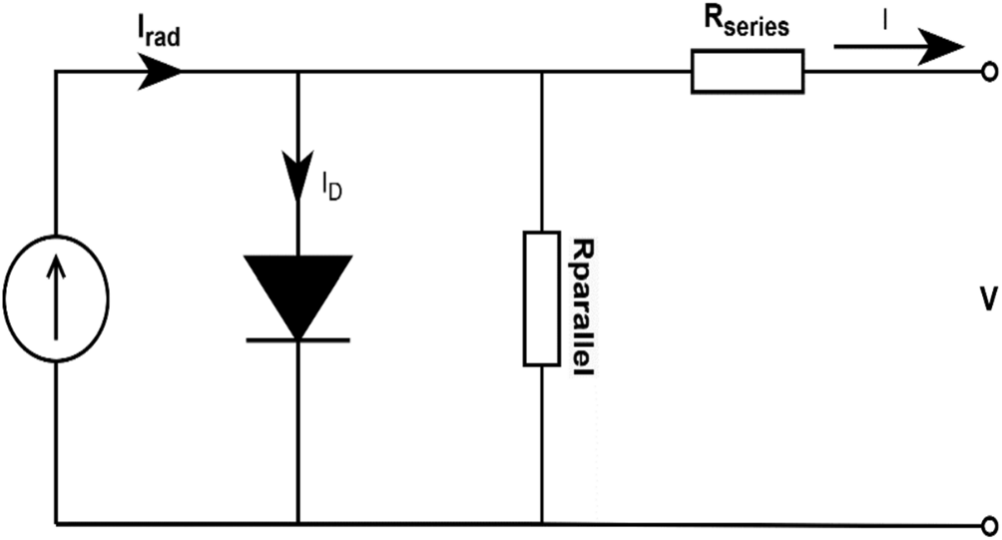
Single diode model of PV module.

A PV module shown in Fig. 8. is formed by combining multiple PV cells to generate DC output. Typically, a current source connected in parallel with a diode comprises the PV circuit. The single diode model is shown in Fig. 8, where, I_rad_ is the photocurrent influenced by the temperature and irradiance the PV cell receives. R_parallel_ and R_series_ are parallel and series resistors, respectively, whereas I_D_ indicates the reverse saturation current. ${V}_{Th1}$ and ${\beta }_{1}$ is thermal voltage and diode-ideality factor respectively.

Mathematically, the output current of PV system Ipv is calculated using Kirchhoff’s Current Law. The single-diode arrangements of photovoltaic model uses a series–parallel configuration of PV modules denoted by series module $({N}_{s})$ and parallel module $({N}_{p}$),. The formula in Eq. (1) controls the PV module’s overall current output I and voltage output V:

1 $$I={I}_{rad} {N}_{p}-{I}_{D1}{N}_{p}\left[\text{exp}\left(\frac{V+NI{R}_{series}}{{\beta }_{1}{V}_{Th1}{N}_{s}}-1\right)\right]-\frac{V+NI{R}_{series}}{N{R}_{parallel}}$$

where $$N= \frac{{N}_{s}}{{N}_{p}}$$

#### P and O algorithm

**Fig. 9:**
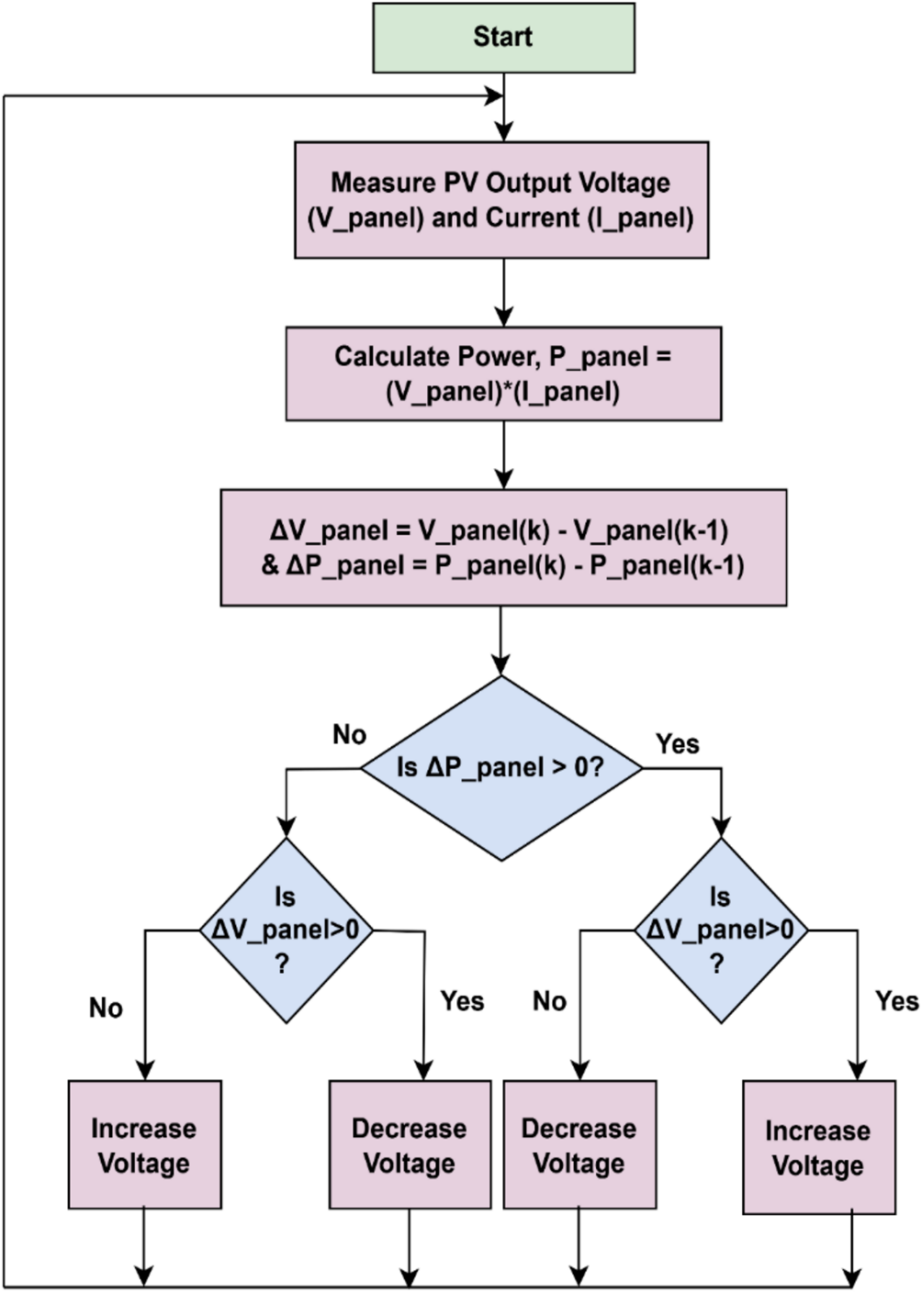
Flow chart of P and O algorithm.

The P &O algorithm depicted in Fig. 9, is the simplest and widely used due to ease of implementation. The basic operation of this algorithm is that it increments and decrement the voltage V_panel and current I_panel to change the power output ΔP. The change in power is observed after perturbation, if the power is increased, the algorithm forwards in same direction and if the power drops, it reverses its direction to move towards maximum power.

#### PSO MPPT algorithm

**Fig. 10:**
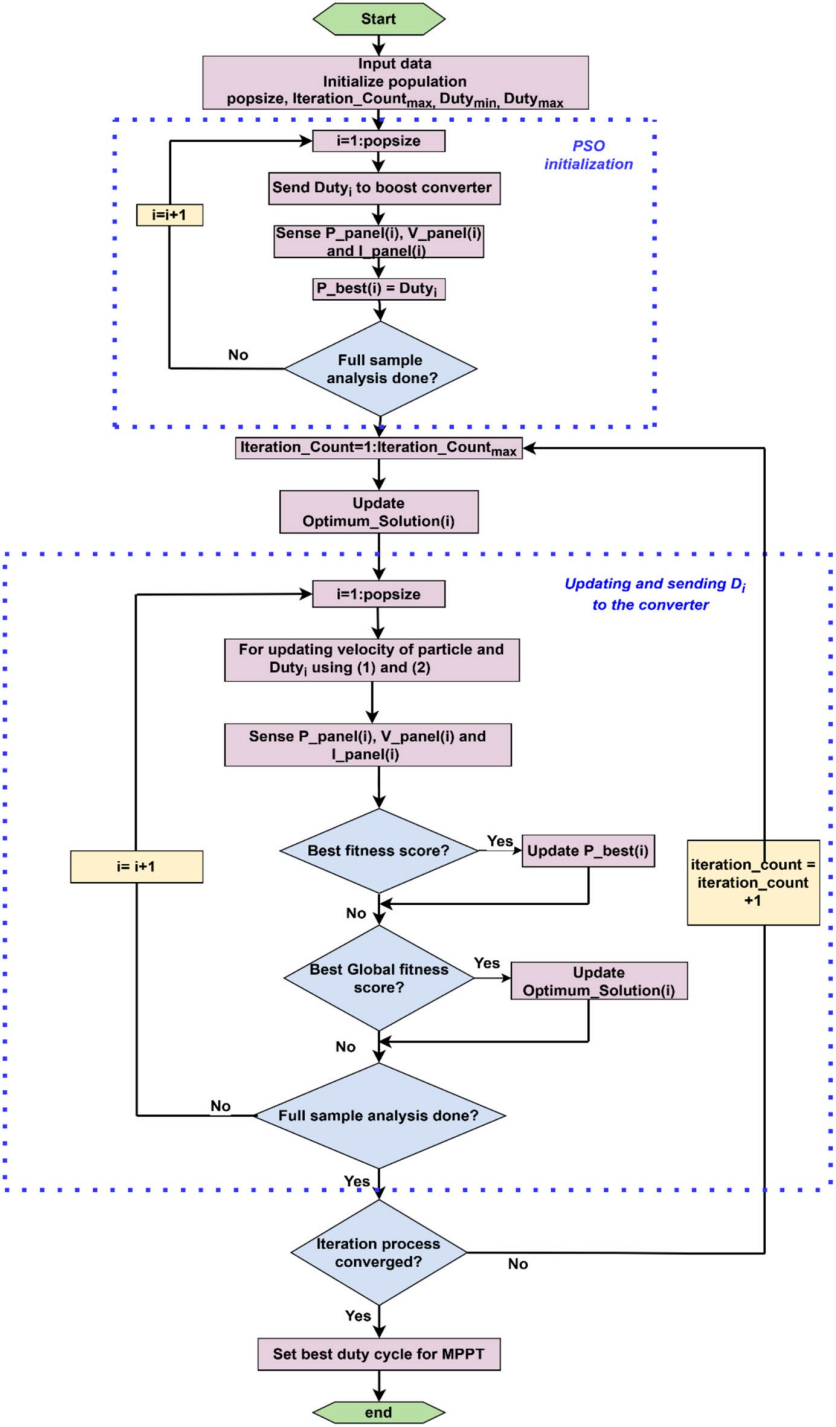
Flowchart of PSO based MPPT.

To compare with P and O, next the PSO tuned algorithm is employed to control the boost converter duty cycle. The PSO algorithm is employed by researchers in various power management strategies^41^. The process to extract the maximum power from the PV module using PSO based method is depicted in Fig. 10. The algorithm begins with the initialization of duty cycles $Dut{y}_{i}$ which are consecutively sent to the boost converter and the power output by each respective values of duty cycle is calculated. The configuration yielding the maximum power with a certain best sample duty cycle is referred as ${Duty}_{best}^{k}$. Update of new duty cycle samples are done by using the following equation which is Levy Flight equation:

2 $${Duty}_{i}^{k+1}= {Duty}_{i}^{k}+{L}_{m}\left(\frac{P}{{\left|Q\right|}^{\frac{1}{\alpha }}}\right) \left({Duty}_{best}^{k}-{Duty}_{i}^{k}\right)$$

where β is the inertia weight which is 1.5, ${L}_{m}$ is the Lévy multiplication factor, and P and Q are randomly selected from the normal distribution function and ${\sigma }_{P}^{2}$ and ${\sigma }_{Q}^{2}$ are the zero mean and variances respectively So,

3 $$P \approx N\left( {0,\sigma_{P}^{2} } \right)\,{\text{and}}\,Q \approx N\left( {0,\sigma_{P}^{2} } \right)$$

The variables ${\sigma }_{P}$ and ${\sigma }_{Q}$ are calculated using the gamma function:

4 $$\sigma_{P} = \left( {\frac{{{\Gamma }\left( {1 + {\upalpha }} \right)*{\text{sin}}\left( {\frac{\pi \alpha }{2}} \right)}}{{{\Gamma }\left( {\frac{1 + \alpha }{2}} \right)*\alpha *\left( 2 \right)^{{\frac{\alpha - 1}{2}}} }}} \right)^{{\frac{1}{\alpha }}} and\,\,\sigma_{Q} = 1$$

The particle velocity can be depicted using the current position ${X}_{i}{\left(t\right)}^{d}$, particle velocity ${V}_{i}{\left(t\right)}^{d}$, and global best position (${G}_{bi}^{d}$) using equation below

5 $${V}_{i}{\left(t+1\right)}^{d}={V}_{i}{\left(t\right)}^{d}\gamma +\left({P}_{bi}^{d}-{X}_{i}{\left(t\right)}^{d}\right)*c{c}_{1}*r{r}_{1}+\left({G}_{bi}^{d}-{X}_{i}{\left(t\right)}^{d}\right)*c{c}_{2}*r{r}_{2}$$

where.

${V}_{i}{\left(t\right)}^{d}$ x provides the i-th particle’s velocity in the d-th dimension at iteration t. It combines the particle’s inertia with the effect of the swarm intelligence’s social and cognitive components to reflect the direction and amplitude of the particle’s movement in the solution space.

${X}_{i}{\left(t\right)}^{d}$ is the current particle (i) position with dimension d at iteration t. It represents the particle’s movement in the solution. It combines its inertia with the influence of the swarm intelligence’s social and cognitive components to reflect the direction and magnitude of the particle’s movement in the solution space.

${P}_{bi}^{d}$ represents the individual particle’s personal best location based on its historical data, which is the optimal duty cycle value discovered thus far in dimension d. It also influences the particle’s future movement by rewarding successful orientations.

${G}_{bi}^{d}$ is the global best position, any particle in the whole swarm in dimension d, finds the best-performing duty cycle. It is the most alluring target during velocity updates, directing the particles toward the ideal MPPT solution.

The acceleration coefficients, or constants $c{c}_{1}$ and $c{c}_{2}$, establish the relative importance of the global best and personal best positions in affecting a particle’s new velocity, with $c{c}_{1}$ stressing individual experience and $c{c}_{2}$ stressing collective intelligence.

Stochastic behaviour is introduced into the particle’s velocity by multiplying the acceleration coefficients by the random values $r{r}_{1}$ and $r{r}_{2}$, which are uniformly distributed between 0 and 1. This prevents premature convergence and ensures diversity in exploration.

#### Particle swarm-optimized + ANFIS-based algorithm for MPPT

To overcome the limitations such as low tracking speed, high steady state error and oscillations (shown in Table 6) compared to P&O and PSO MPPT techniques, a hybrid PSO + ANFIS based MPPT algorithm is proposed. The steps involved in this algorithm are:

1. Training data generation

To train the ANFIS-based MPPT controller, a synthetic dataset was created using standard photovoltaic modelling equations. A total of 1000 samples were generated by varying solar irradiance (G) from 0 to 1000 W/m^2^ and temperature (T) from 15 °C to 35 °C.

2. ANFIS structure and membership function design

A Sugeno-type fuzzy inference system (FIS) was initialized using MATLAB’s genfis3, which applies fuzzy C-means clustering. Ten clusters were chosen, resulting in ten fuzzy rules. Each input variable was represented using triangular membership functions (MFs), chosen for their simplicity and computational efficiency. The MF parameters were initialized based on the cluster centroids.

3. Rule base formation

The rule base was automatically generated by genfis3 function.

4. PSO-based parameter optimization

##### PSO parameters

Table 3 parameters are used to train the ANFIS controller. PSO was utilized to optimize the FIS structure. The particles were used to represent a candidate set of FIS parameters. These parameters were iteratively tuned by the algorithm to reduce the prediction error between target values and the output of the FIS.

**Table 3:** Parameters used by PSO for ANFIS optimization.

Parameters	Value
Swarm size	25
No. of iterations	20
Inertia weight (w)	1 (damped by 0.99)
Personal coefficients	1
Social coefficients	2
Particle position limits	[−25, 25]

PSO setup comprised of 25 swarm size particles, 20 iterations, and adaptive inertia weight. Optimization was done to both input membership functions and the constants of the FIS output.

The best particle’s parameters were applied to the ANFIS, resulting in an optimized MPPT controller capable of tracking the maximum power point with high accuracy and robustness.

5. Performance metrics

The trained controller was validated on test data.

The following important MATLAB functions were employed in the implementation:

- LoadData: Loads and splits the PV dataset.
- CreateInitialFIS: Creates the sugeno-type FIS by using clustering.
- GetFISParams and tSetFISParams: Retrieves and sets FIS parameters for optimization.
- TrainFISCost: Declares the root mean square error-based cost function employed by PSO.
- RunPSO and TrainAnfisUsingPSO: Run the PSO algorithm and control the training process.
- Evalfis: Evaluates the trained fuzzy system.
- PlotResults: Plots predicted vs. actual output and error trends.

**Fig. 11:**
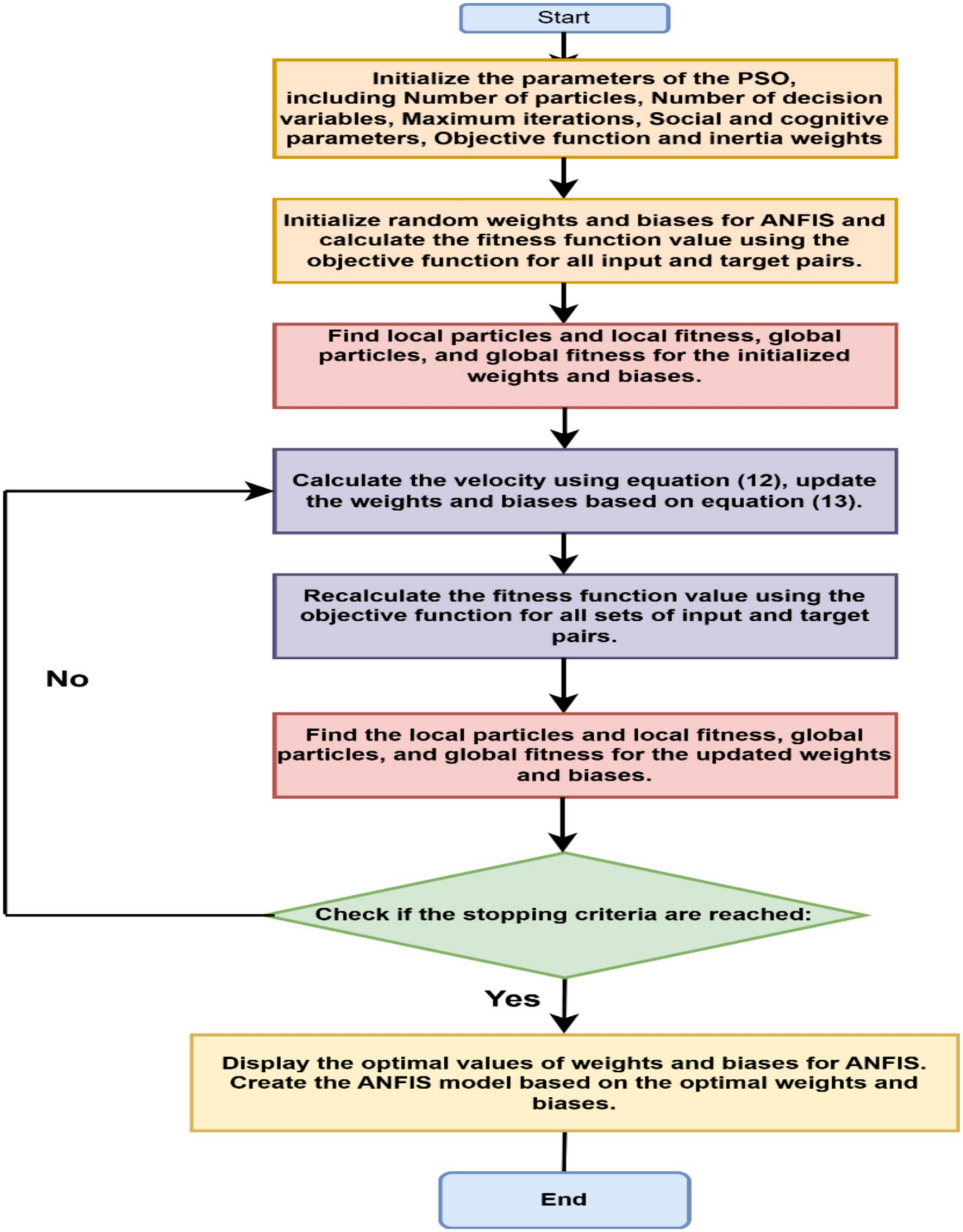
Flowchart of PSO + ANFIS algorithm.

To implement this system, the PSO + ANFIS controller algorithm organizes a five layer layered architecture model, the algorithm is shown in Fig. 11. The input layer 1 of the algorithm is the fuzzyify layer in which the crisps inputs are converted into fuzzy values using MFs.

6 $${O}_{pq}^{1}=\mu \left({I}_{pq}^{1}\right)$$

The layer 2 is known as fuzzification layer which applies the fuzzy AND operation by using the product function. This layer performs the activation of fizzy rules.

7 $${O}_{i}^{2}={\omega }_{i}=\prod\nolimits_{p=1}^{u}{O}_{pq}^{1}$$

The Layer 3 is the normalization layer in which the fuzzy rule values are actively normalized.

8 $${O}_{i}^{3}=\overline{{\omega }_{i}}= \frac{{O}_{i}^{2}}{{\sum }_{m=1}{y}^{2} {O}_{n}^{2}}$$

The layer 4 is the de-fuzzify layer in which linear function is applied to the parameters that are trainable and fine tunes the non-linear parameters.

9 $${O}_{i}^{4}=\overline{{\omega }_{i }}\left({d}_{1i }{I}_{1}^{1}+{d}_{2i}{I}_{2}^{1}+\dots +{d}_{yi}{I}_{y}^{\left(1\right)}+{d}_{0}\right)$$

The layer 5 is the output layer which produces an output by adding up all the inputs. The summation of weighted linear output gives the single value output.

10 $${U}_{a}={o}^{5}=\sum\nolimits_{m=1}^{{y}^{2}}{O}_{i}^{4}= \sum\nolimits_{m=1}^{{y}^{2}}\overline{{\omega }_{i}}{f}_{i}= \frac{\sum_{m=1}^{{y}^{2}}{\omega }_{i}{f}_{i}}{\sum_{m=1}^{{y}^{2}}{\omega }_{i}}$$

The model accuracy is produced by the formula below. The Mean Square formula is:

11 $$MSE=(1/n )\sum\nolimits_{k}{\left|{t}_{k}-{O}_{k}\right|}^{2}$$

The model performance is measured by mean squared error (MSE), which is calculated as the mean squared difference between target and expected output values. The PSO algorithm minimizes the MSE in a network with outputs. PSO simulates a swarm of particles, each of which is a potential solution, to optimize the network parameters. To reduce the MSE and improve the model’s accuracy, the particles iteratively update their positions in the search space using both the global best solution and their individual best performance.

The velocity and location are expressed mathematically by Eqs. (12) and (13) respectively.

12 $${V}_{k}{\left(t+1\right)}^{s}={V}_{k}{\left(t\right)}^{s}\gamma +\left({P}_{bk}^{s}-{X}_{k}{\left(t\right)}^{s}\right)*C{C}_{1}*R{R}_{1}+\left({G}_{bk}^{s}-{X}_{k}{\left(t\right)}^{s}\right)*C{C}_{2}*R{R}_{2}$$

13 $${X}_{k}{\left(t+1\right)}^{s}={V}_{k}{\left(t+1\right)}^{s}+{X}_{k}{\left(t\right)}^{s}$$

**Fig. 12:**
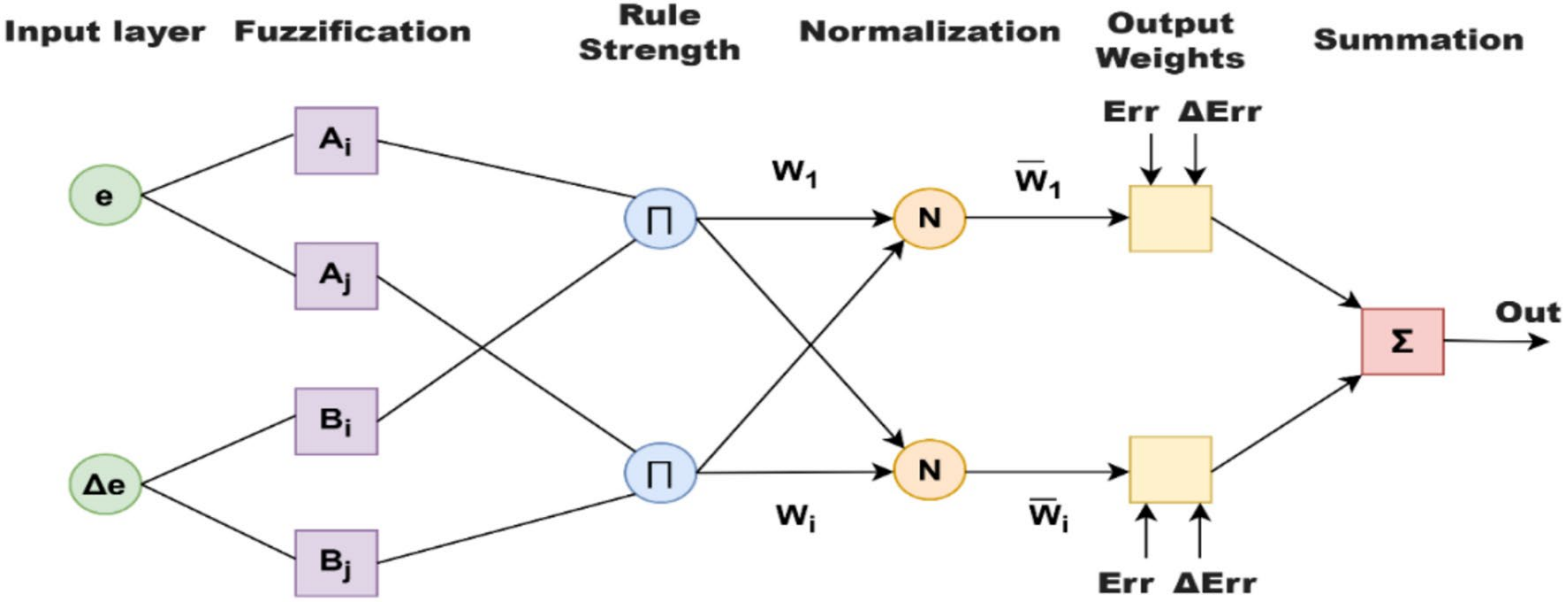
The ANFIS layered architecture.

**Fig. 13:**
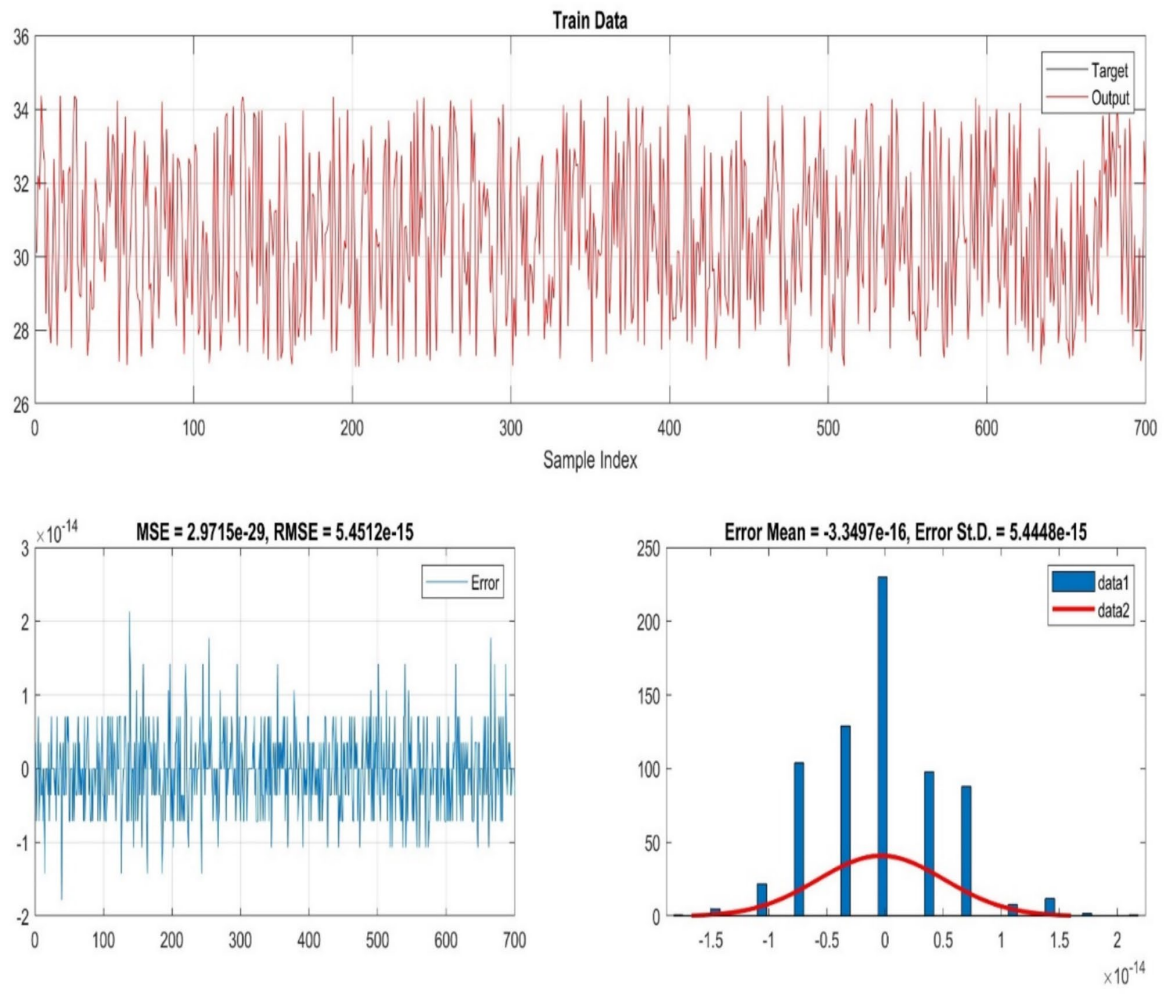
Train and test data for ANFIS.

Figure 12. show the architecture of the ANFIS model employed in this research. The structure clearly illustrates the five-layer design, which involves fuzzification, rule evaluation, normalization, defuzzification, and output layers, all of which combined allow the system to learn from data and make smart decisions. Subsequent to this, Fig. 13. illustrates the ANFIS model training and test data performance, proving that the system was able to achieve accuracy in measuring the nonlinear mapping between input and output variables. The fact that the training curve closely follows that of the testing proves the excellence and generalizability of the proposed ANFIS method.

### Design of DAB controller

With its inherent bidirectional power flow and isolation at high frequencies, the DAB converter is at the core of controlling energy exchange among PV, battery, and grid. However, its nonlinear dynamics, load disturbances, and parameter variation pose difficulties for traditional PI or linear controllers, which usually lack adequate transient response and are prone to oscillation. To address these shortcomings, a fuzzy controller is deployed to offer adaptive, rule-based decision-making, characteristic of the system’s nonlinear and time-variant nature.

**Fig. 14:**
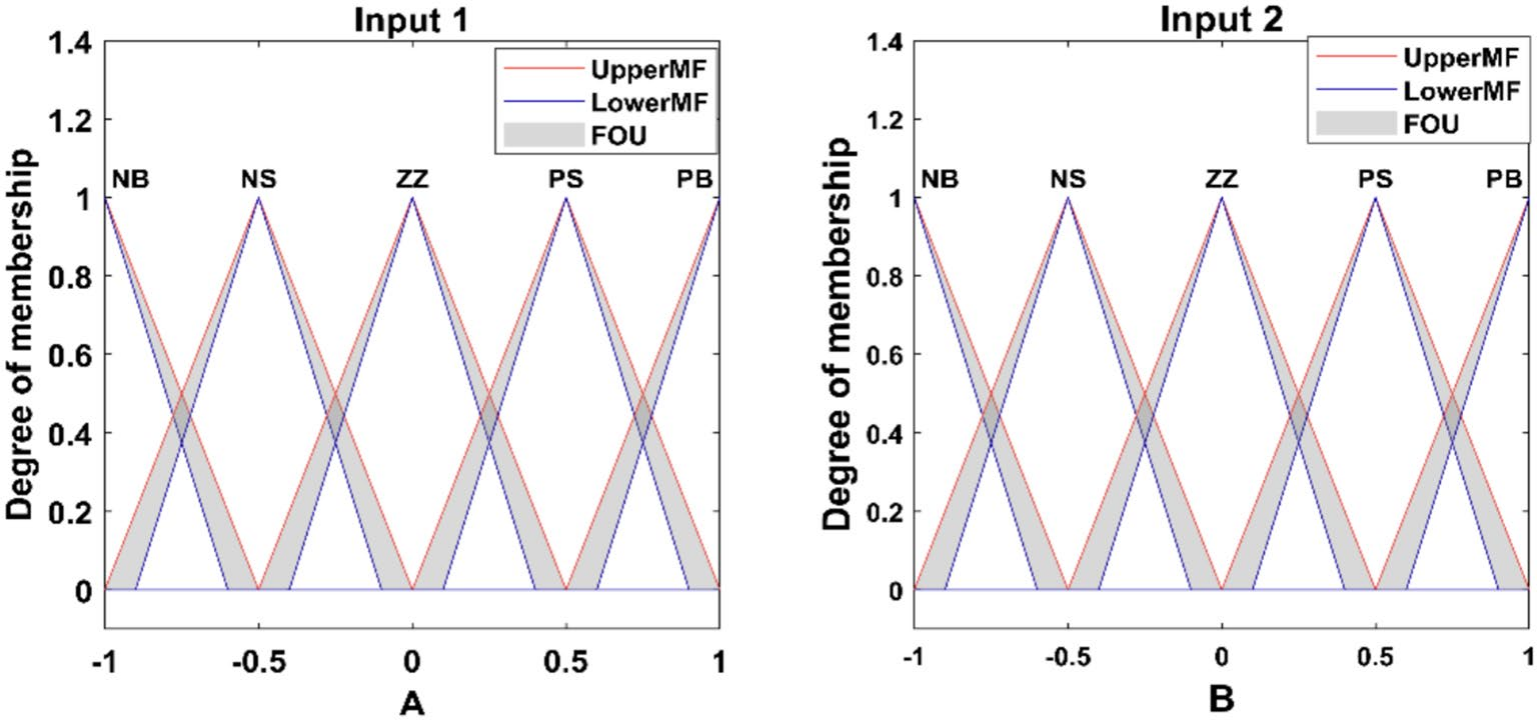
Fuzzy type-2 controller for DAB.

The fuzzy control strategy also follows the overall intelligent architecture of the paper, with MPPT using ANFIS to implement intelligent control over both power transfer (DAB) and power extraction (PV). Figure 14 shows the type-2 fuzzy controller diagram, which uses the sugeno type membership function to calculate the phase shift output value for the better dynamic performance of the controller. In this research, a type-2 fuzzy logic controller was used in place of traditional type-1 fuzzy and predictive control techniques because of its better capability to deal with uncertainty in the environmental state. Unlike type-1 fuzzy, where it is considered that membership functions are crisp, type-2 fuzzy have a FOU, which enables the system to handle measurement noise, sensor uncertainty, and sudden changes in irradiance or temperature more efficiently.

The controller maps pairs of these inputs to optimized phase shift outputs, allowing intelligent modulation of power flow. The 25-rule matrix depicted in Table 4 is the knowledge base of the fuzzy controller.

**Table 4:** Fuzzy rule base used in the fuzzy (type-2) controller.

Error (A)/Change in error (B)	NB	NS	Z	PS	PB
NB	S	S	M	M	B
NS	S	M	M	B	VB
Z	M	M	B	VB	VB
PS	M	B	VB	VB	VVB
PB	B	VB	VB	VVB	VVB

NB: Negative Big, NS: Negative Small, Z: Zero, PS: Positive Small, PB: Positive Big, S: Small, M: Medium, B: Big, VB: Very Big, VVB: Very Very Big.

### Switching modes of the proposed PV-assisted EV charging system with vehicle-to-grid (V2G) integration

#### G2V mode

When solar power from the PV array is not available, the conventional AC grid is the only source of energy for both AC household loads and charging the EV. This AC power goes through a source converter initially, which converts the AC supply from the grid to DC. The DAB converter follows this, which reduces the voltage to meet the needs of the EV battery. When the PV system produces power, it is in parallel with the AC grid to supplement the energy load. The DC power produced by the PV is supplied into the system by the source converter and DAB converters, minimizing the use of grid power. Here, the source converter controls power diversion and DC-link voltage regulation and is used to ensure proper voltage scaling for charging the battery by the DAB^42,43^. If an EV is not plugged in for charging, the PV energy available is directed to the AC load. Here, the source converter functions as an inverter, converting the DC output of the PV into AC to feed the load directly, thus decreasing the grid load.

#### V2G Mode

When PV power is not available and the EV is plugged in with a high enough SoC, the EV becomes an energy source with backup capability. The DAB converter boosts the voltage from the battery to the desired level of DC-link, and the source converter converts this DC power into AC, enabling the EV to recharge power back to the grid to supply local AC loads. When both the PV and the EV battery are present (for instance, during the day), the system gives priority to energy from these two sources to satisfy the AC load requirement. Energy from the EV and PV is regulated through the DAB and the source converter as needed. In case the combined supply from the above two sources is not enough, the rest of the power is fed automatically from the AC grid. When only the PV system is operational (no EV plugged in), the solar energy available is utilized to feed the AC load via the source converter.

## Results

### Hardware-in-loop (HIL) setup for real-time validation of the proposed system

**Fig. 15:**
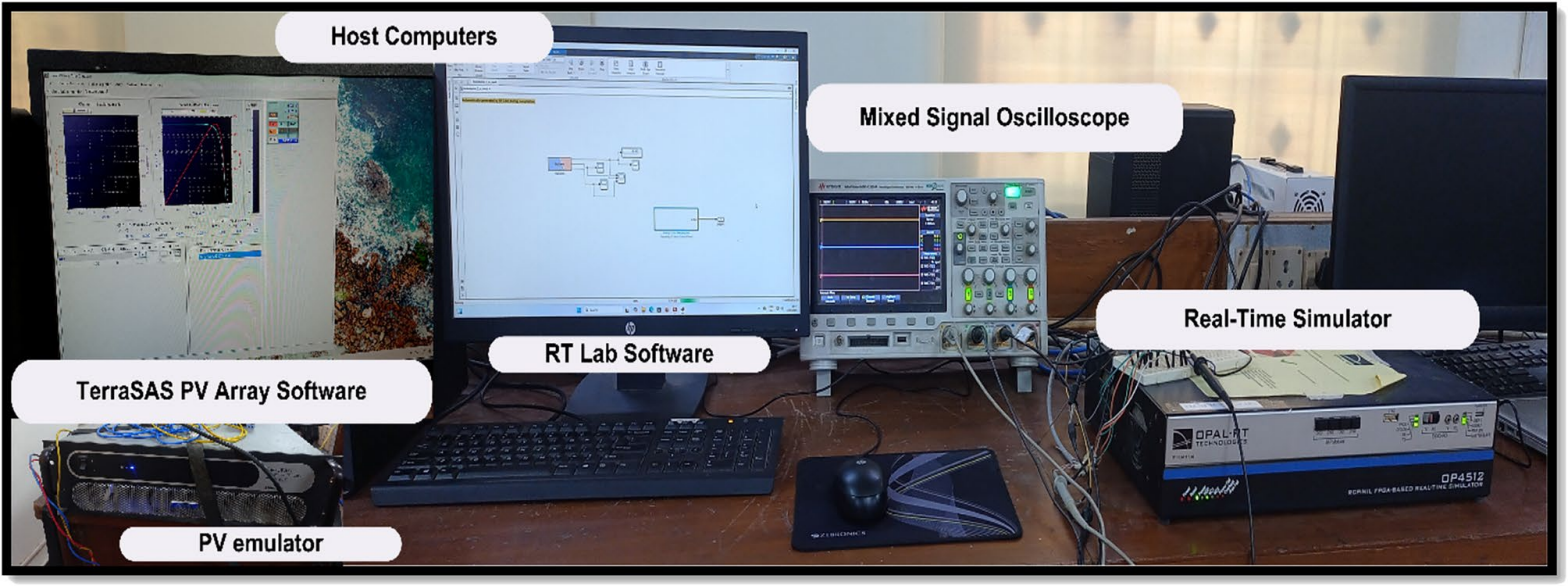
Experimental setup for real-time validation.

**Fig. 16:**
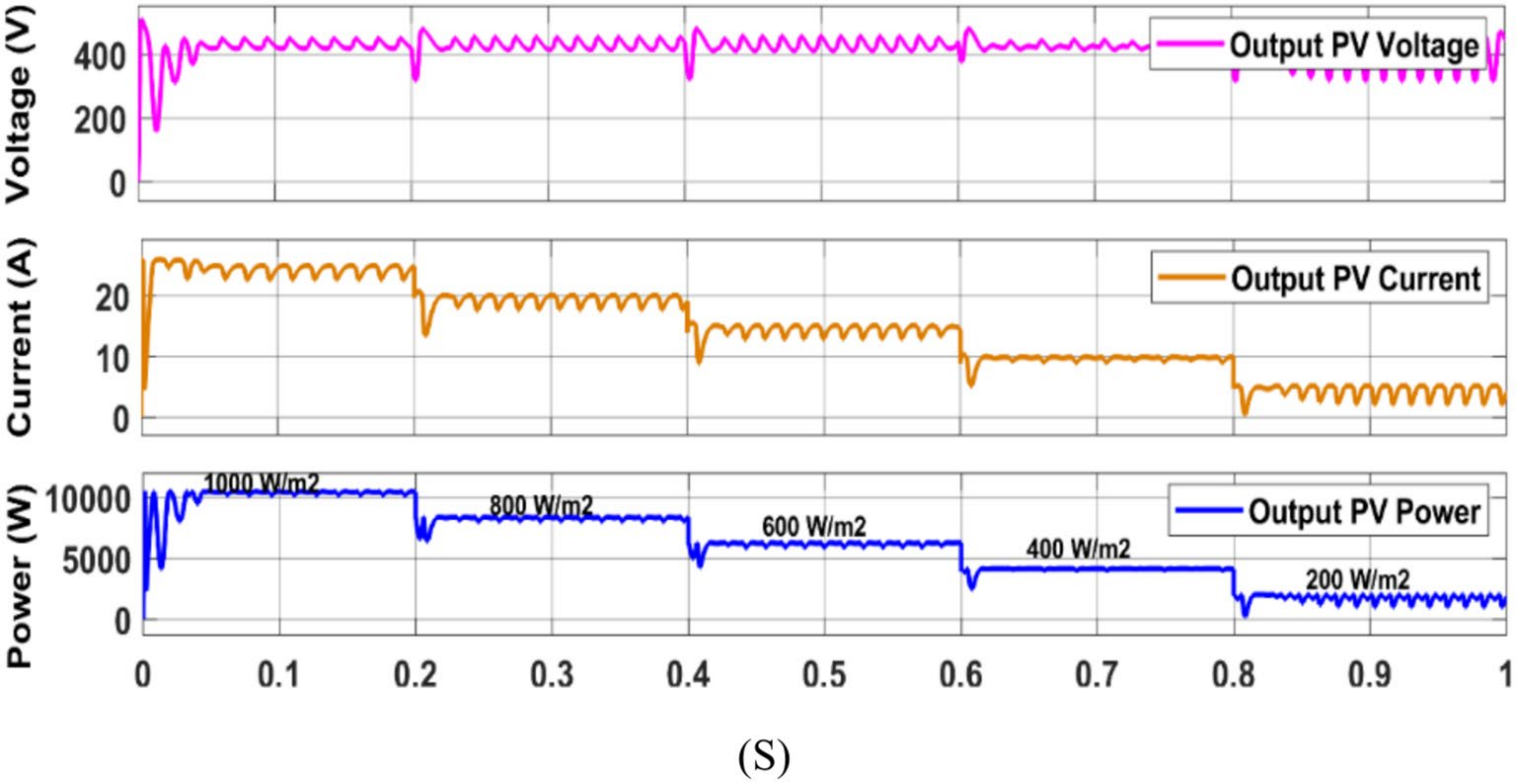
Simulation (S) result of PV module output employing P and O MPPT.

**Fig. 17:**
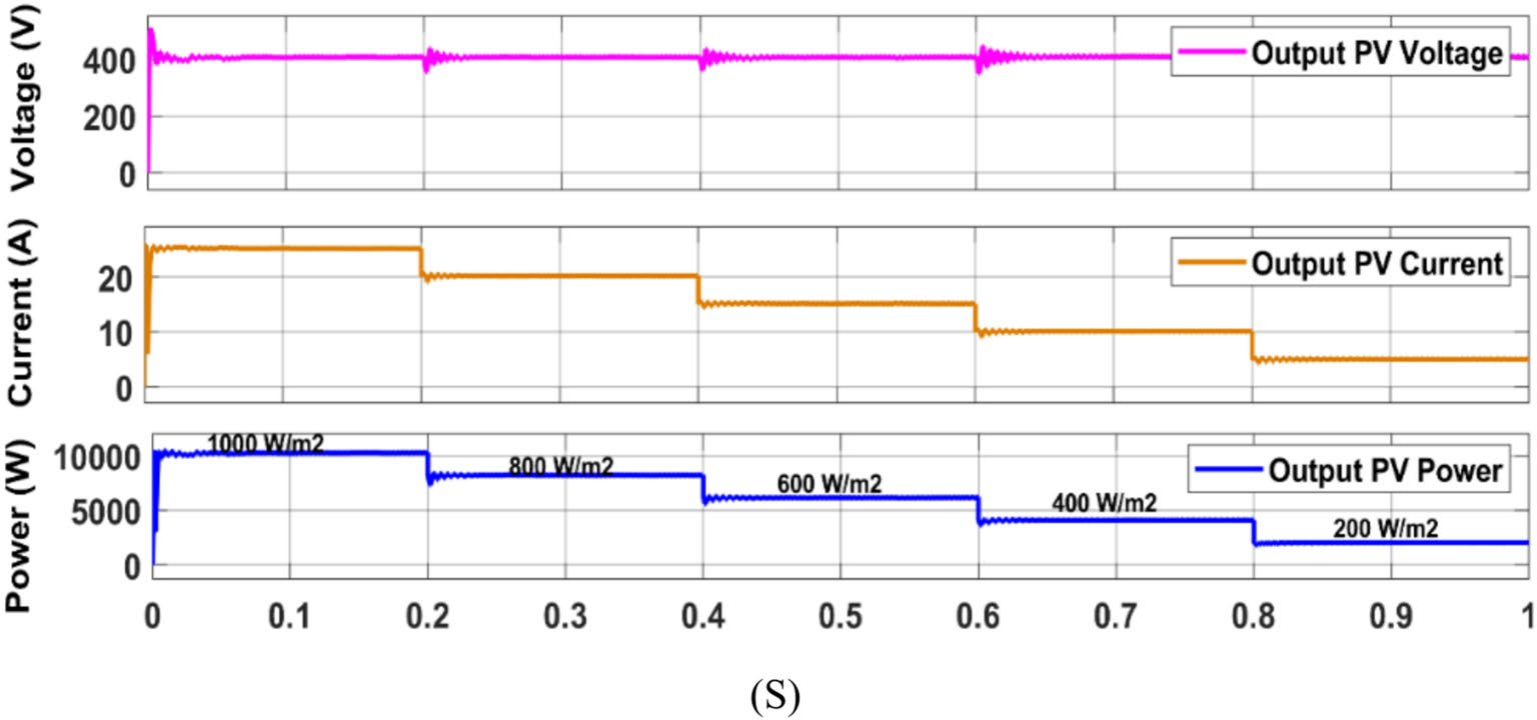
Simulation (S) result of PV module output employing PSO based MPPT.

**Fig. 18:**
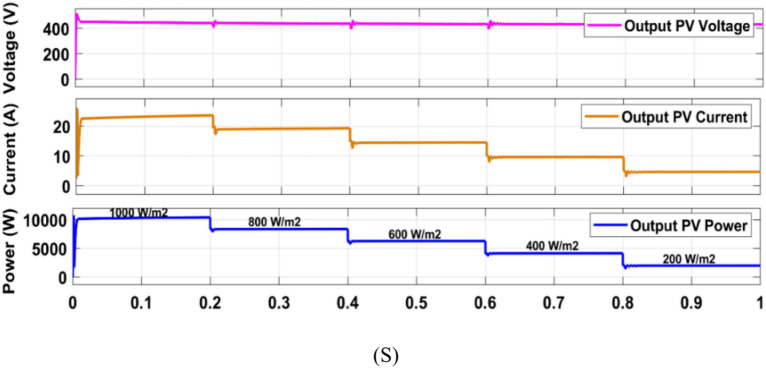
Simulation (S) result of PV module output employing PSO + ANFIS MPPT.

**Fig. 19:**
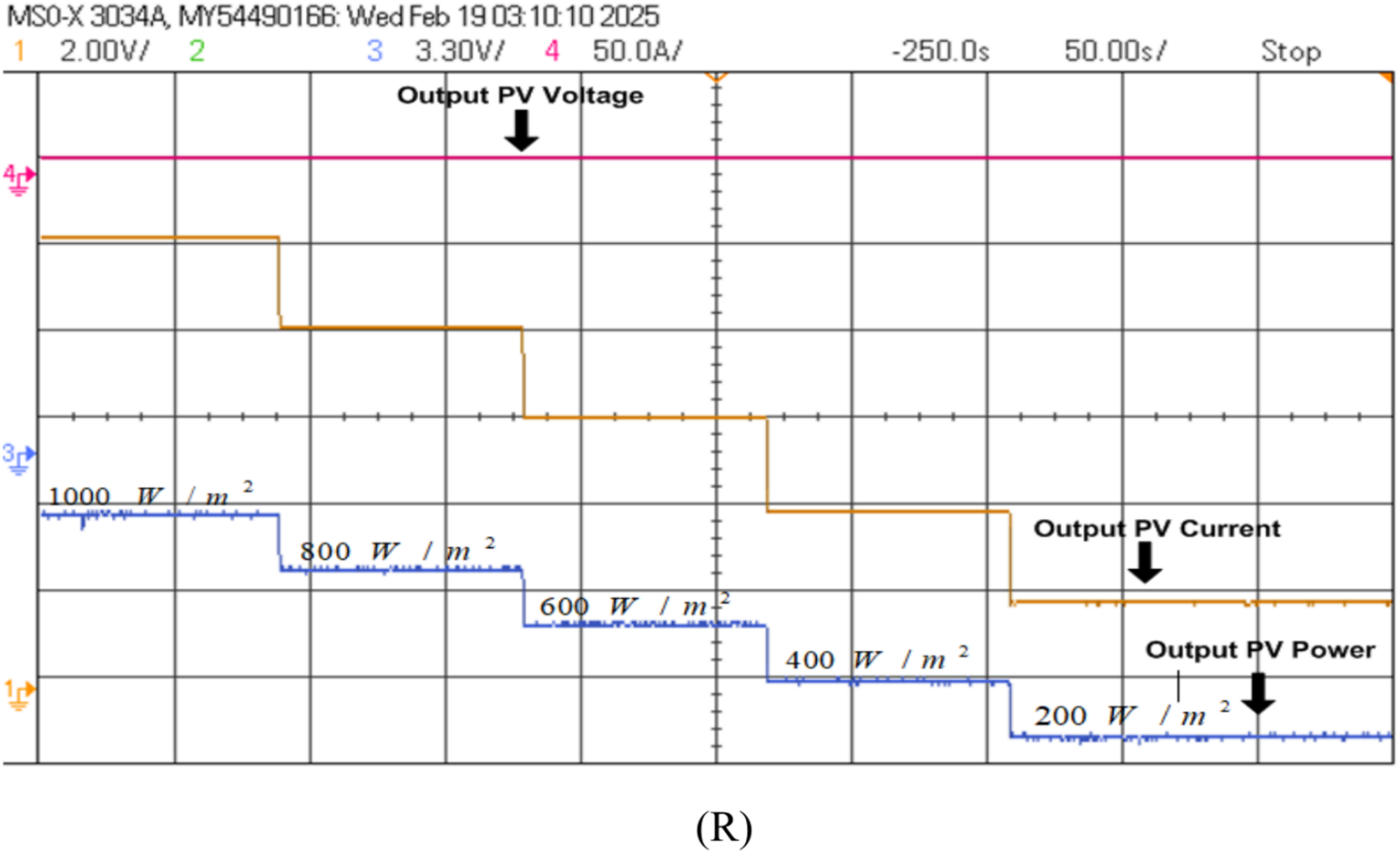
HIL (R) result of PV module output employing PSO + ANFIS MPPT.

**Fig. 20:**
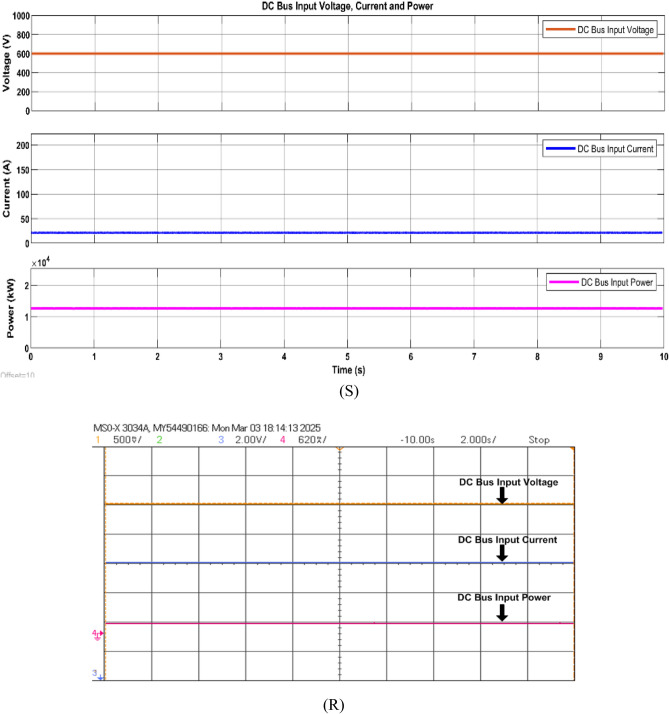
Simulation (S) and HIL (R) result of DC bus voltage current and power.

**Fig. 21:**
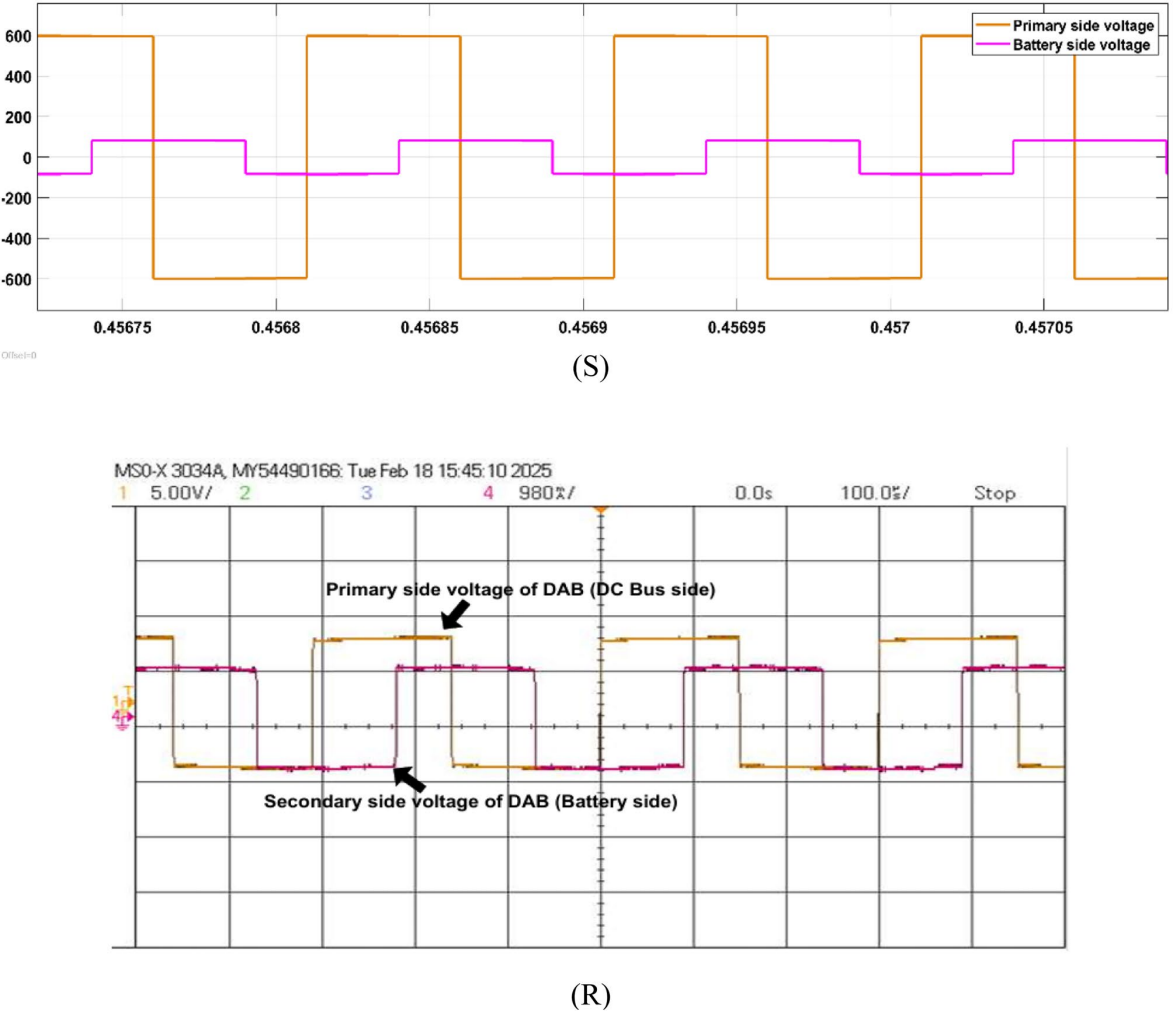
Simulation (S) and HIL (R) results of DAB primary and secondary voltage.

**Fig. 22:**
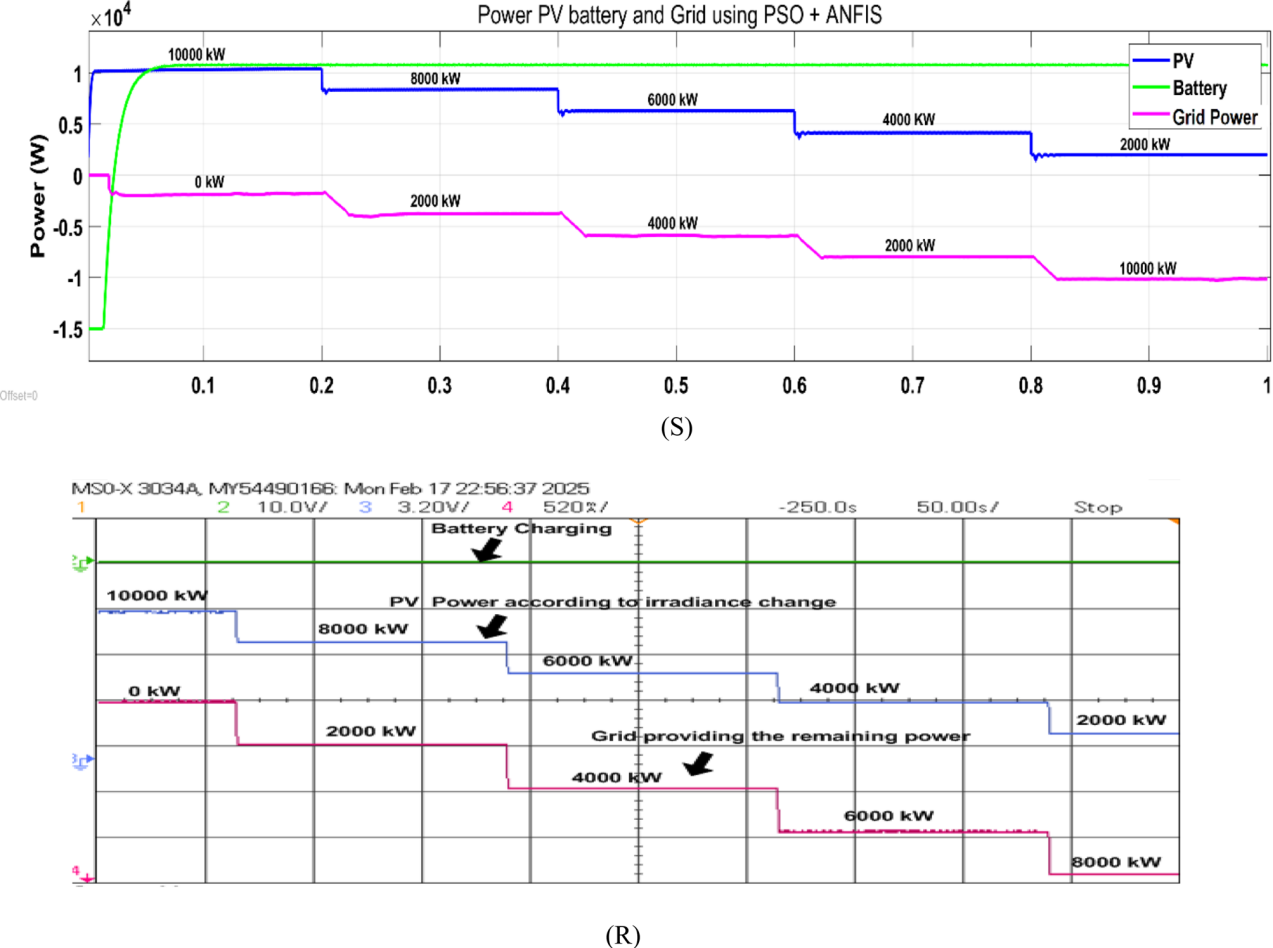
Simulation (S) and HIL (R) result of grid providing remaining power to charge the battery using PSO + ANFIS MPPT.

**Fig. 23:**
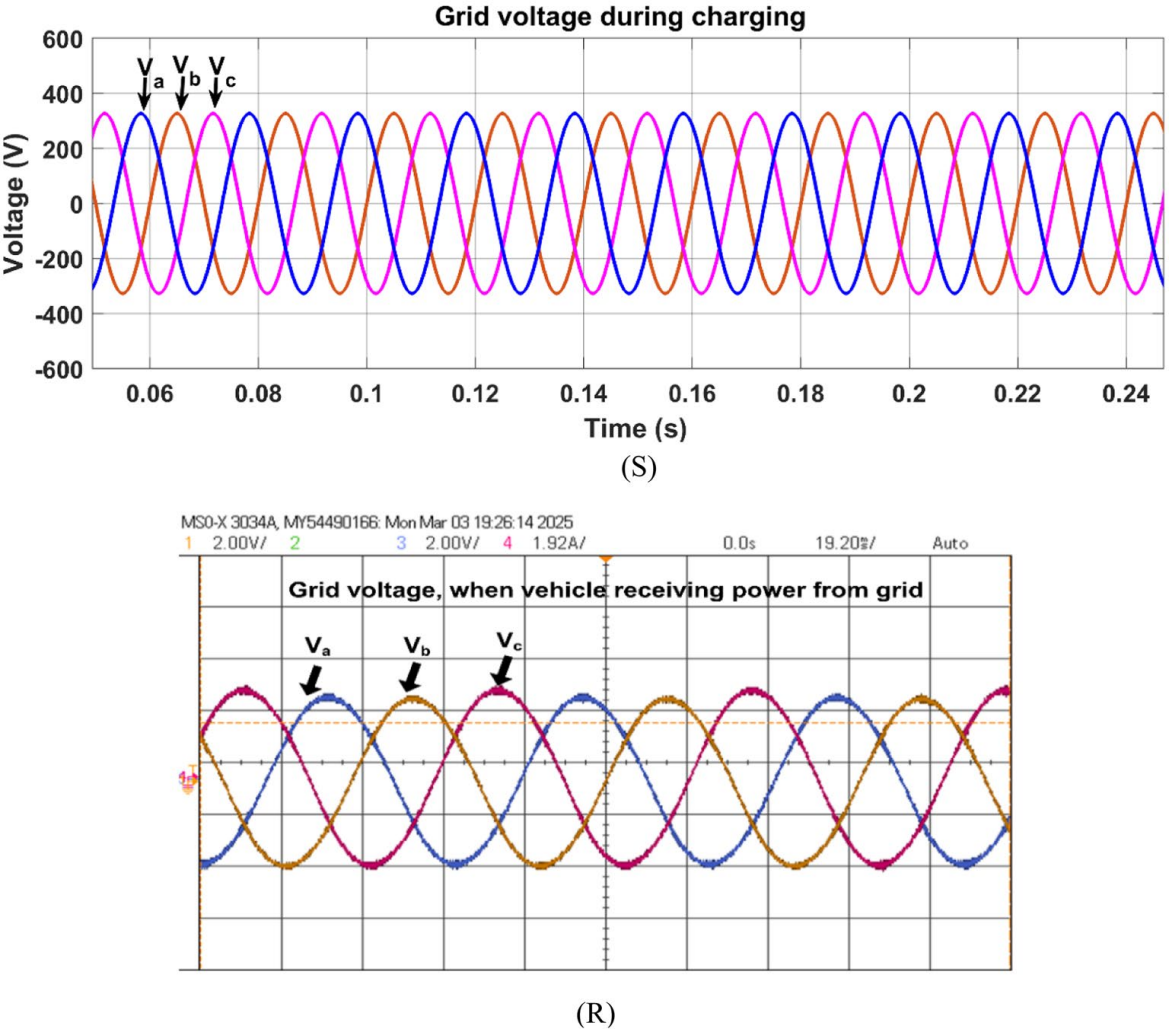
Simulation (S) and HIL (R) result of grid voltage during charging mode.

**Fig. 24:**
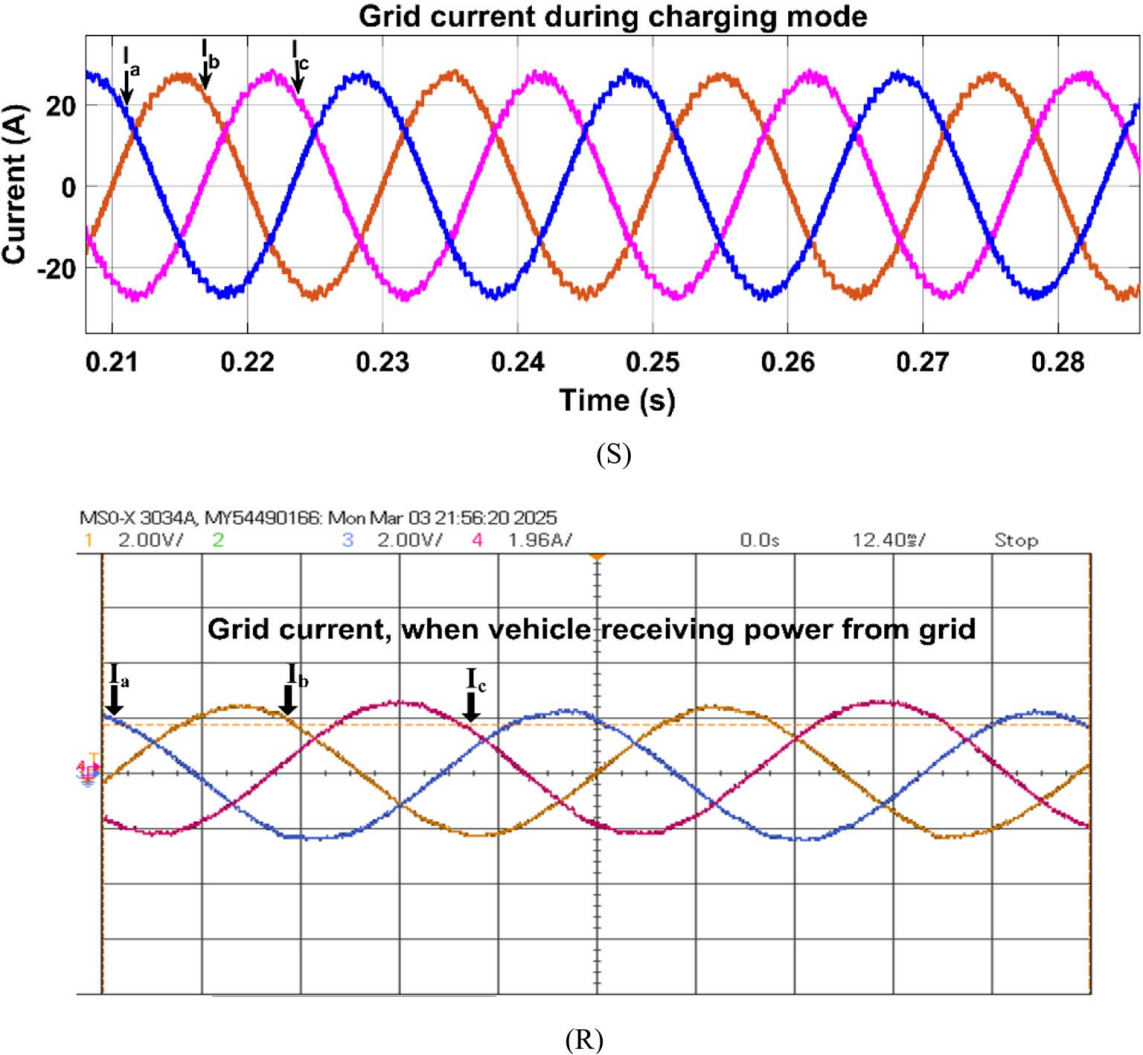
Simulation (S) and HIL (R) result of grid current in charging mode.

**Fig. 25:**
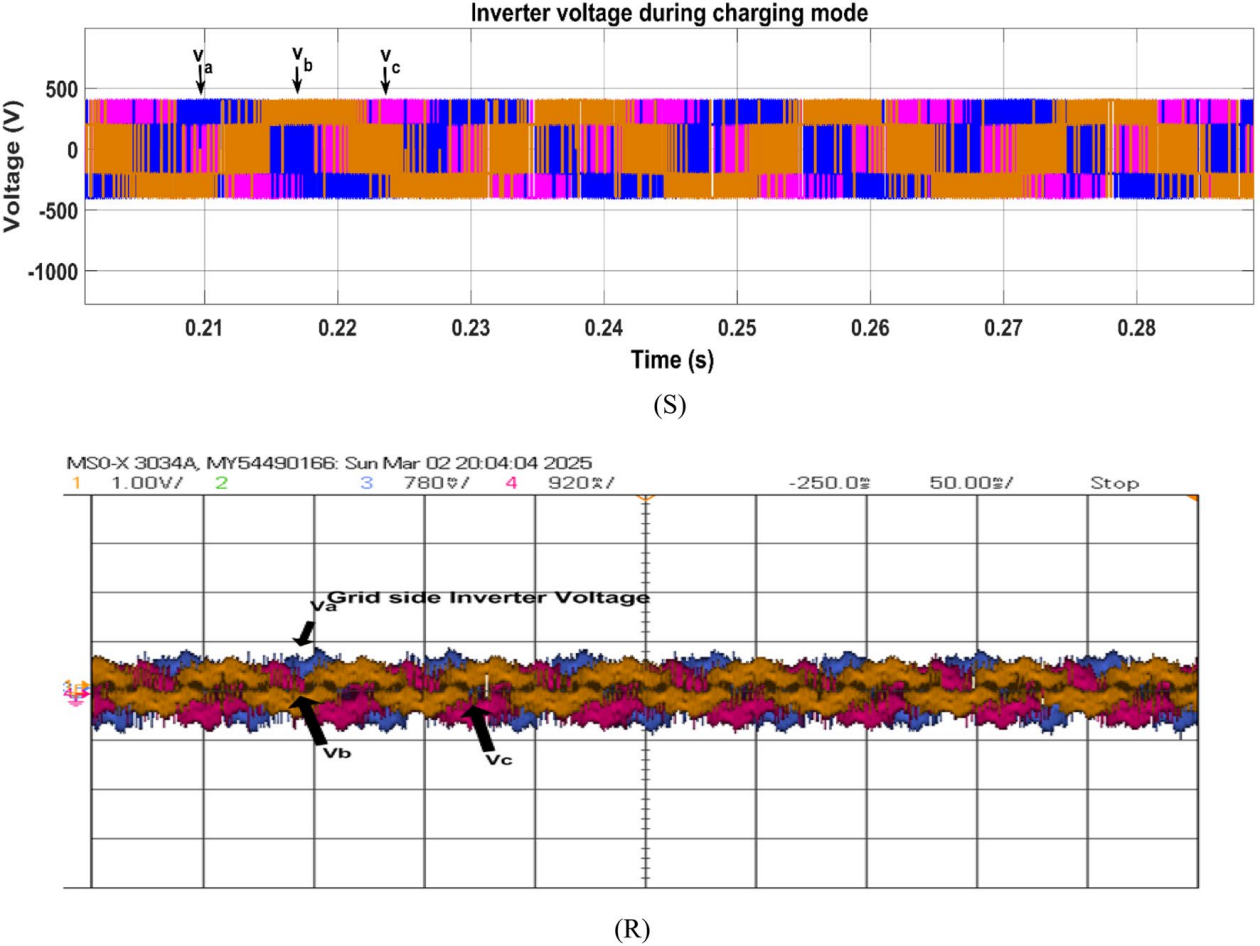
Simulation (S) and HIL (R) result of grid inverter voltage during charging mode.

**Fig. 26:**
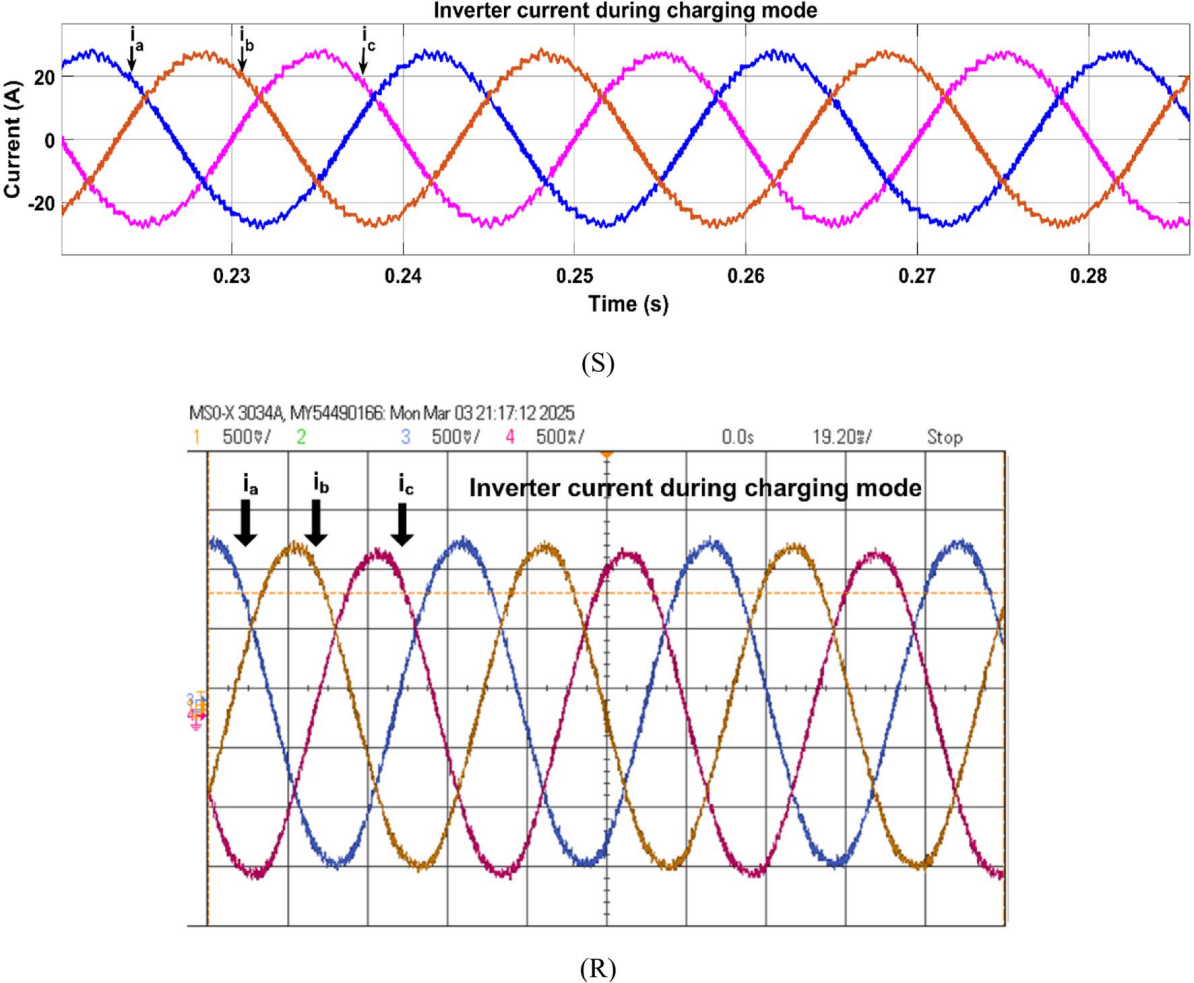
Simulation (S) and HIL (R) result of inverter current during charging mode.

**Fig. 27:**
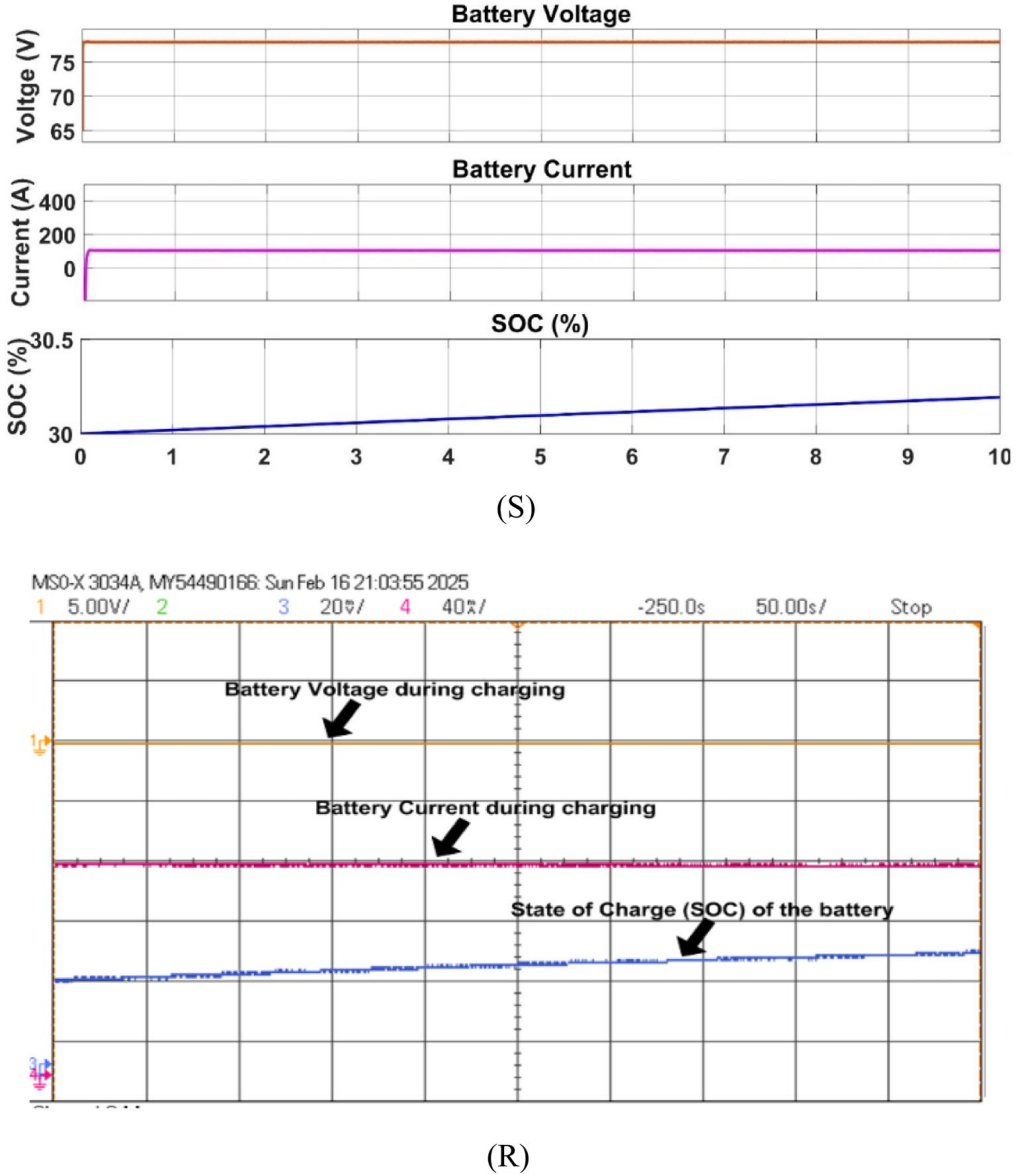
Simulation (S) and HIL (R) result of battery during charging mode.

**Fig. 28:**
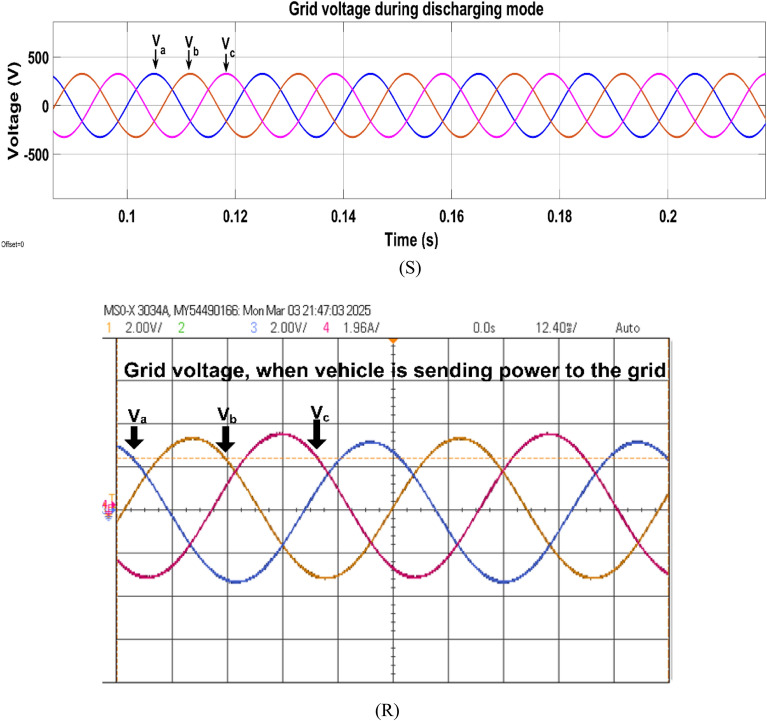
Simulation (S) and HIL (R) result of grid voltage during discharging mode.

**Fig. 29:**
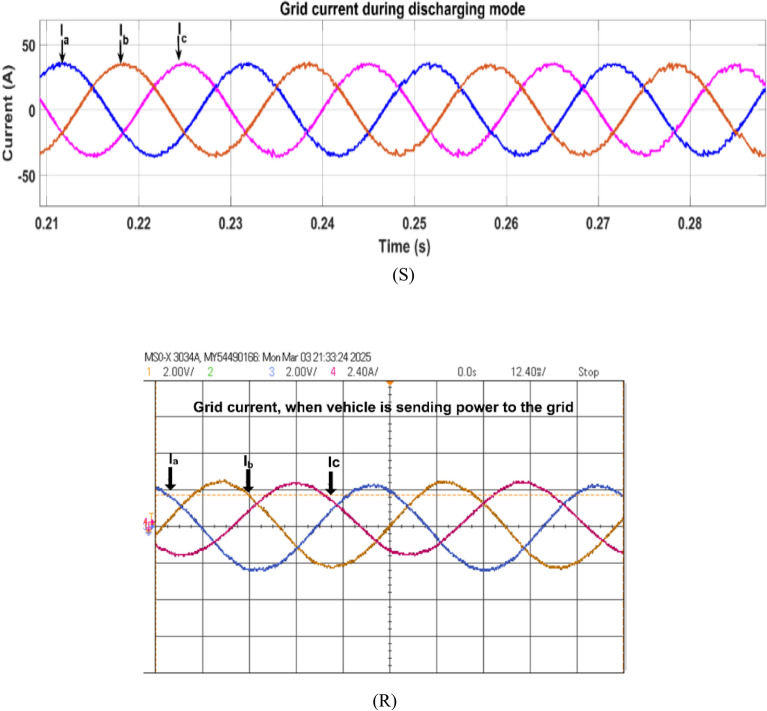
Simulation (S) and HIL (R) result of grid current during discharging mode.

Firstly, the system simulation is carried out in MATLAB Simulink and then entire system is validated in a real-time environment on RT Lab, where HIL Test is performed using an FPGA-based Opal-RT simulator. Figure 15 shows the experiment setup to carry out HIL validation in the lab. Table 5 shows the OPAL-RT’s specifications for experimenting. In Fig 16-29 ‘S’ represents the MATLAB Simulation results, and ‘R’ represents the HIL validated results obtained using OPAL-RT It is observed that the real-time results matches closely with the simulation results, validating the reliability of the suggested control under real-time conditions.

**Table 5:** Specifications of the Opal-RT simulator.

**Device name**	Opal-RT
FPGA	EHSX32
Input output lines	Analog 16 channels, digital 32 channels
Input- output connectors	4
Monitoring connectors	Rear DB37 connectors
Computer interface	4 optional, 5 Gbps SFP optical interfaces

### MPPT performance analysis

In this work (Fig. 1), the system begins with a PV emulator (600 V, 16 A) that provides a DC power based on irradiance. MPPT algorithms are employed to track the maximum power at various irradiances. Three algorithms to track MPP are used here and simulation results are given below (Figs. 16, 17, 18). A comparative analysis of these methods is shown in Table 6. The analysis yields that PSO + ANFIS hybrid method leads to highest tracking efficiency therefore it performance is tested in HIL and then same is used in further proposed work of charging and discharging.

**Table 6:** Comparison table of P and O, PSO and PSO + ANFIS algorithm.

Parameter	P and O	PSO	PSO + ANFIS
Tracking speed	0.86 s	0.44 s	0.29 s
Complexity	Easy	Medium	Highly complex
Steady state error	3.8%	1.4%	0.43%
Efficiency	90%	96%	99%
Oscillations	1.6%	0.5%	0.05%

Figure 19 shows the HIL implementation of PV panel output voltage & current, and power implementing PSO + ANFIS hybrid MPPT method. The change in these values are corresponding to the change in solar irradiance.

The hybrid PSO + ANFIS algorithm attained the tracking efficiency of 99%, with steady-state error of 0.05%, as verified by simulation as well as real-time HIL experiment (Fig. 19). This proves to be applicable for real-time systems with fluctuating irradiance, where traditional schemes like P&O show reduced response and more oscillations.

Next, a boost converter is fed with the PV power whose duty cycle is controlled using proposed MPPT algorithms for the extraction of maximum power. The boost converter steps up the voltage from PV to the DC Bus level (600 V) and maintains the same. Figure 20 shows the stable DC voltage, current and power at DC bus. This system is further integrated trough DC bus with DAB and grid for bidirectional power flow management.

Next, the DAB converter, linked with DC bus, provides the bidirectional power flow between the DC bus and the EV battery. The DAB in the system is responsible for allowing the charging of the battery from the PV panel and/or from the grid and also supplying the power back to the grid when excess power is available from PV or battery. Here, a single-phase shift control method along with fuzzy type-2 controller is used to enhance the converter performance. Figure 21 shows the DAB converter’s primary (DC bus side) and secondary voltage (battery side).

### Charging mode (G2V) operation

The DAB converter is connected to both a lithium-ion (Li-ion) battery and the AC grid. Specifications of the battery are given in Table 7. Charging of the battery is carried out in two modes: PV mode and grid mode. In the present case, the initial SOC of the battery is 30%. The source of charging (PV or grid) is dynamically selected based on the real-time availability of PV and grid power. The AC grid supplies AC power, which is converted into stable DC power by a source converter before being delivered to the common DC bus to facilitate reliable energy flow for battery charging and overall system operation.

**Table 7:** Li-ion battery parameters.

Parameters	Rating
Nominal voltage of the battery	72 V
Rated capacity in kWh	10 kWh
Initial SOC (%)	30%

During this operation, the EV battery is supplied with power from PV source and any shortfall in this is compensated by the grid as shown in Fig. 22.

Figure 23 shows the three-phase voltages (V_a_, V_b_, V_c_) of the grid during the charging mode. The controlled voltage (600 V) regulates at the DC bus through the AC-DC source converter irrespective of change in grid or load conditions. This ensures smooth power transfer and uninterrupted charging as the grid voltage is stable.

The grid current is in 180° phase shift with the grid voltage when grid is supplying power to the vehicle battery as shown in Fig. 24. The phase currents are I_a_, I_b_ and I_c_ respectively.

The voltage of the inverter remains sinusoidal with the grid voltage due to the phase locked loop control ensuring the proper operation. Figure 25 shows the grid side inverter voltage.

In G2V mode, the inverter current is 180° out of phase with the grid inverter voltage, which confirms that the grid is supplying the power. The LCL filter smoothened the ripples of the grid current; the waveforms can be observed in Fig. 26.

The battery is charged in G2V mode, drawing 10 kW from the grid, the battery charging is shown with initial SoC of 30% as presented in Fig. 27.

The G2V mode operated stably under dynamic conditions. The DC bus voltage kept itself stable at 600 V during PV-grid power transfer, while the voltage and current waveforms of the inverter remained sinusoidal and phase-locked to the grid. HIL verification ensured that power supply remained continuous even when PV output varied, demonstrating the adaptive ability of the suggested control strategy.

### Discharging mode (V2G) operation

In V2G mode, the battery power is sent to the grid; the battery delivers 10 kW power to the grid; the bidirectional DAB helps to draw the power from the battery and send it to the grid; the DAB works in boost mode during V2G conversion as the voltage is stepped up from 72 to 600 V. Figure 28 shows the three phase grid voltage when the vehicle is sending power to the grid.

The grid current is shown in Fig. 29, here grid current is in phase with the grid voltage, representing vehicle battery feeding the grid.

**Fig. 30:**
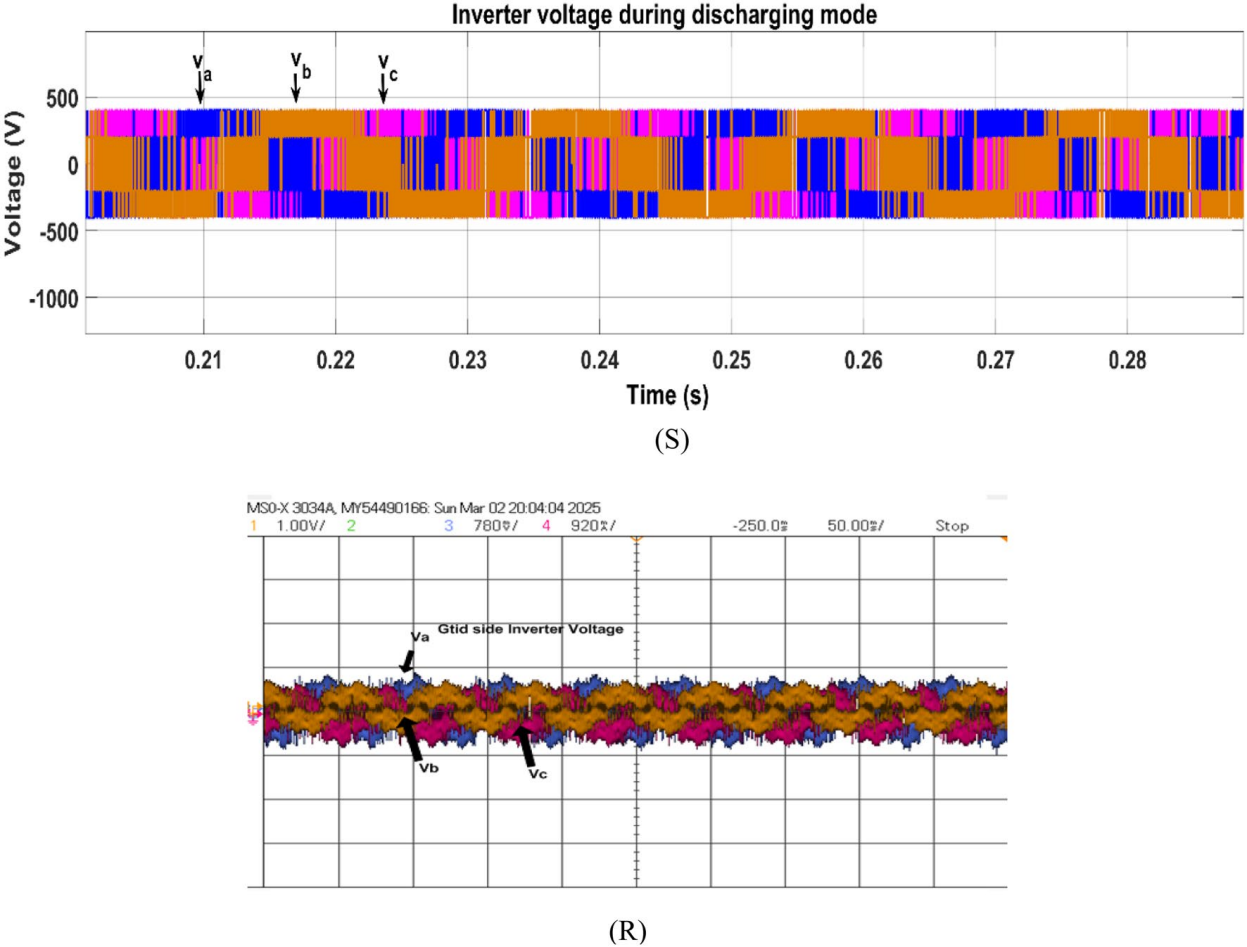
Simulation (S) and HIL (R) result of inverter voltage during discharging mode.

**Fig. 31:**
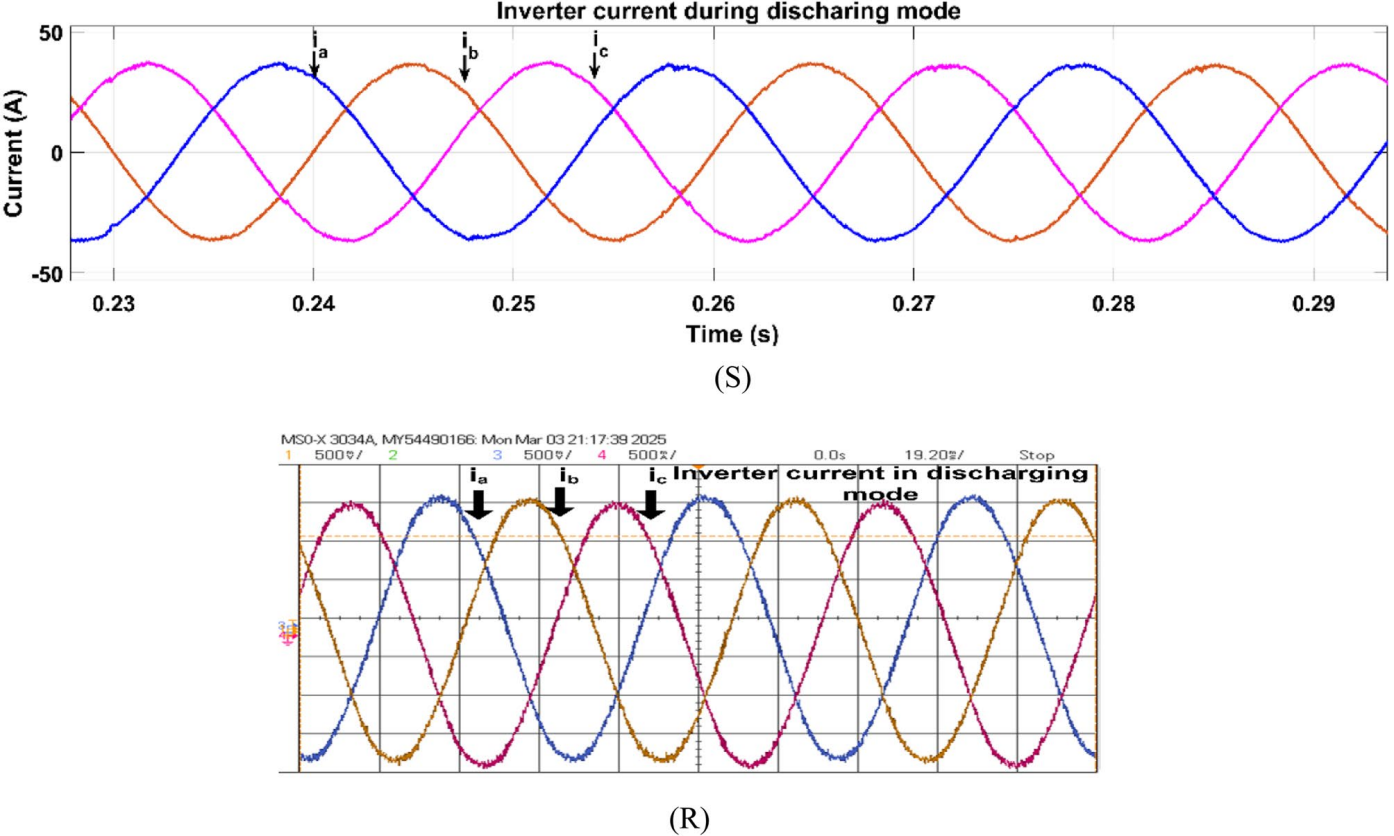
Simulation (S) and HIL (R) results of inverter voltage during discharging mode.

Figure 30 shows the inverter voltage in V2G mode. The inverter voltage remains sinusoidal and synchronized with the grid voltage in V2G mode. Figure 31 illustrates the inverter current waveform in V2G mode which is sinusoidal aligned with grid voltage which shows the real power injecting from battery to grid.

**Fig. 32:**
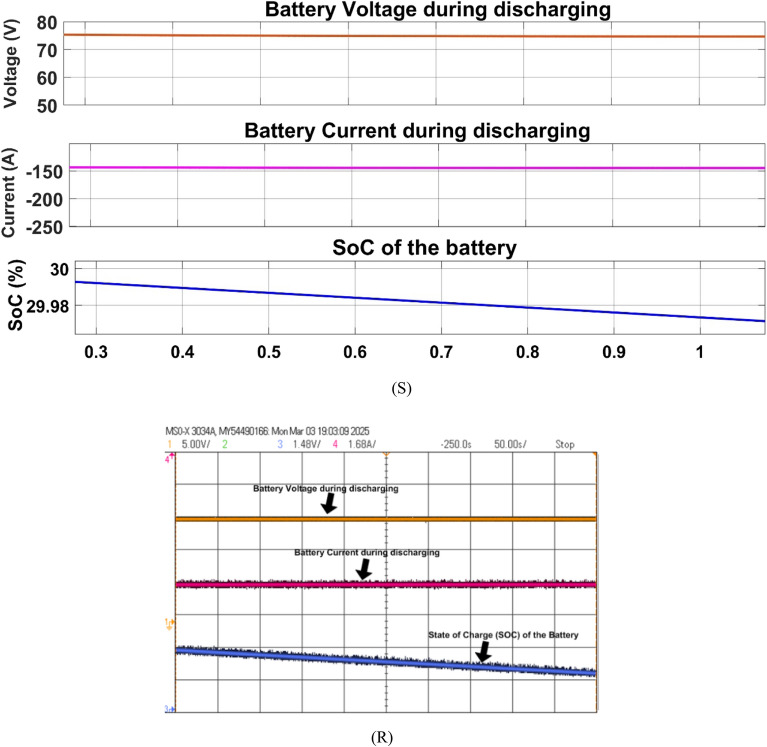
Simulation (S) and HIL (R) result of battery during discharging mode.

Figure 32 depicts the battery voltage current and SoC during the discharging mode (V2G), the battery supplies 10 kW power to the grid while discharging.

During discharge mode (V2G), the battery actually delivered power to the grid. The DAB converter was in the boost mode for boosting the battery voltage from 72 to 600 V DC, and the inverter had sinusoidal waveforms in synchronization with the grid. The in-phase grid current (Fig. 29) validates successful injection of power. Simulation and HIL results both show the viability of this bidirectional control under real-time operation.

### MPPT performance of PV during PSC

**Fig. 33:**
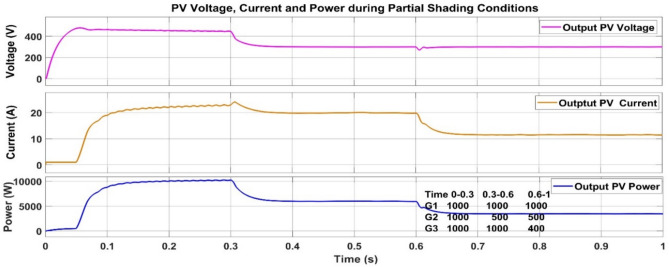
PV voltage, current and power during the PSC.

**Fig. 34:**
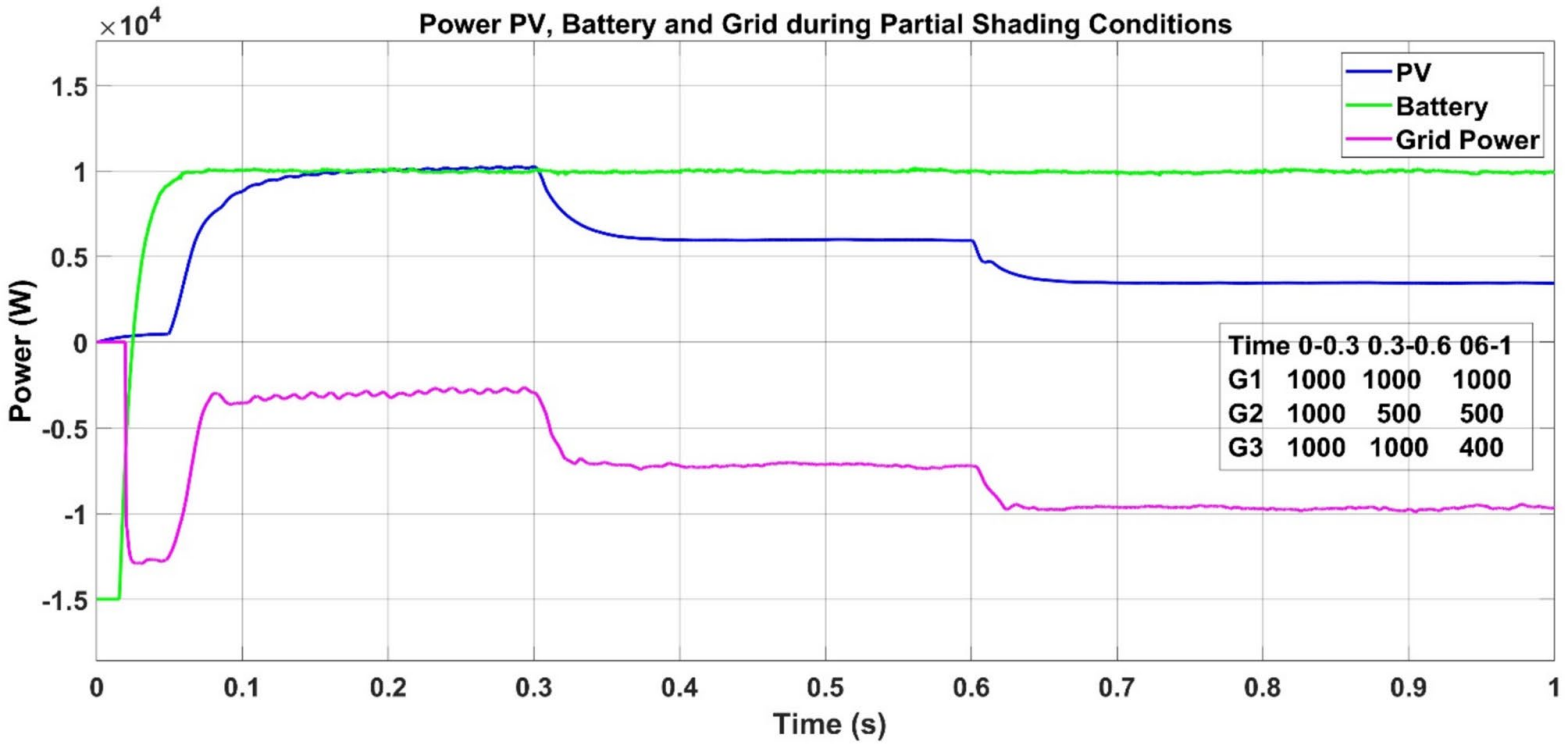
Simulation result of grid providing remaining power to charge the battery using PSO + ANFIS MPPT during PSC.

In order to assess the performance and the reliability of the suggested MPPT algorithm in real environmental conditions, a dynamic irradiance profile was simulated on a three-panel PV array. The test sequence for PV voltage, current and power during PSC is shown in Fig. 33 and starts with a uniform irradiance of 1000 W/m^2^ over all panels for the first 0–0.3 s period, simulating the best scenario. At 0.3 s, a shading event of partial shade is created by decreasing the irradiance on panel G2 to 500 W/m^2^ while G1 and G3 are still fully illuminated, representing a mild non-uniform situation. In the last step, between 0.6 and 1.0 s, the shade intensity increases as G3 falls even further to 400 W/m^2^, producing a more complicated power-voltage curve with several local maxima. This simulated PSC profile is created to test the controller’s capability to track the global maximum power accurately and operate efficiently under rapidly changing weather conditions. The PSC profile is tested for all the three of P and O, PSO and PSO + ANFIS algorithm, the results are shown in Table 8. Similarily Fig. 34 shows the grid power utilization to charge the battery using PSO + ANFIS MPPT during PSC.

**Table 8:** Comparison table of P and O, PSO and PSO + ANFIS algorithm during the PSC.

Parameter	P and O	PSO	PSO + ANFIS due to PSC
Tracking speed	1.3 s	0.82 s	0.28 s
Complexity	Easy	Medium	Highly complex
Steady state error	3.8%	1.4%	0.8%
Efficiency	90%	96%	99%
Oscillations	1.6%	0.5%	0.05%

The proposed PSO + ANFIS algorithm outperforms P&O and PSO in both normal and partial shading conditions. While performance indices slightly reduce in PSC as expected. The controller still achieves fast convergence (0.40 s), high efficiency (97.5%), and extremely low oscillations (0.12%), showing its stability under time-varying irradiance.

## Conclusion

The proposed work present switching the design, modelling, and real-time validation of a 10 kW bidirectional EV charging/discharging system combining PV and grid sources to achieve clean and efficient energy management. The system lessens the shortcomings of conventional fossil fuel-based EV charging through a hybrid source architecture using dynamic switching between PV and grid. The vehicle is primarily charge through PV power but in case of high demand it includes/shifts to grid power. PSO-based ANFIS hybrid MPPT technique is employed to yield stable and efficient solar energy extraction under changing irradiance. The comparative analysis of various MPPT algorithms is given in Table 5 and Figs. 16, 17, 18, which observes that the PSO-based ANFIS approach exhibits a maximum tracking efficiency of 99.5%, notably greater than conventional P&O and PSO techniques. A sugeno fuzzy (type 2) controller is developed to control the DAB converter for bidirectional power flow management and DC bus voltage stability, facilitating seamless switching between G2V and V2G modes.

The proposed architecture shows promising scalability to larger PV arrays. Its modular structure makes it possible to extend the control scheme to centralized or multi-string PV topologies using system-specific data to retrain the controller. Yet, practical implementation is challenged by several issues. To start, type-2 fuzzy inference’s higher computational complexity could necessitate optimization for embedded devices like FPGAs or digital signal processors (DSPs). Second, precise irradiance and temperature sensing over distributed arrays becomes increasingly important in the case of partial shading conditions. Third, real-time requirements, communication latency, and hardware restrictions need to be tackled to provide low-latency control action. In addition, easy integration with grid-connected inverters and adherence to smart grid standards will become vital for large scale deployment.

The proposed system is first simulated in the MATLAB/Simulink environment and then validated in real-time on an OPAL-RT-based HIL platform. The results confirm that, PV is first utilized in charging EV batteries, however in case of non-availability of PV power, it automatically switches to the grid supply. PV can also feed to the gird in case of excess PV power and demand from grid. In addition, in the V2G mode, the system can supply stored battery power back to the grid which can be utilized to address peak demand. Using of a fuzzy-controlled DAB converter makes stable and efficient bidirectional energy transfer feasible. The proposed intelligent hybrid charging system exhibits high accuracy, adaptability, and robustness under dynamic operating conditions and is highly suitable for next-generation EV penetration. The integration of PV source and minimized use of the grid contribute to lesser carbon footprints and green & sustainable transportation.

This research has some limitations which need to be recognized. HIL testing was performed on a controlled small-scale system with a single EV interface, and it doesn’t perfectly capture the complex behaviour of a real multi-EV charging environment. An ideal battery (constant capacity, no thermal limits, ideal charging/discharging) is also assumed by the model without accounting for battery degradation or thermal effects, which might affect long-term system performance. Besides, the configuration was all done in a simulation–HIL environment, without any actual hardware elements like physical inverters, chargers, or PV panels. For further research, the system can be expanded to HIL testing under PSC profile. Addition of real-time electricity pricing and demand response would further enhance the controller’s relevance in smart grid scenarios. In addition, experimental testing with physical hardware is to be done to assess robustness of the control system under realistic conditions.

## Data Availability

All data generated or analysed during this study are included in this manuscript.

## Abbreviations

EV: Electric vehicles
PV: Photovoltaic
MPPT: Maximum power point tracking
V2G: Vehicle-to-grid
P & O: Perturb and observe
PSO: Particle swarm optimization
PSO + ANFIS: PSO + adaptive neuro-fuzzy inference system
DC: Direct current
AC: Alternating current
HIL: Hardware-in-loop
PSC: Partial shading conditions
G: Irradiance
T: Temperature
G2V: Grid-to-vehicle
